# Clustering of Unhealthy Lifestyle Behaviours and Its Contextual Determinants in Adolescents: A Multilevel Analysis of School-Based Surveys in 45 Countries

**DOI:** 10.3390/nu17213388

**Published:** 2025-10-28

**Authors:** Yohannes Tekalegn Efa, David Roder, Zumin Shi, Ming Li

**Affiliations:** 1 Cancer Epidemiology and Population Health Research Group, Allied Health and Human Performance, University of South Australia, Adelaide 5000, Australia; david.roder@unisa.edu.au; 2 South Australian Health and Medical Research Institute (SAHMRI), Adelaide 5000, Australia; 3 Department of Nutrition Sciences, College of Health Sciences, QU Health, Qatar University, Doha P.O. Box 2713, Qatar; zumin@qu.edu.qa; 4 Faculty of Medicine, University of Queensland, Brisbane 4006, Australia

**Keywords:** lifestyle, physical activity, dietary habits, smoking, alcohol drinking, clustering pattern, adolescents

## Abstract

**Background:** This study examined the clustering of unhealthy lifestyle behaviours and their determinants among adolescents across Europe, Central Asia, and North America. **Methods:** The study included 210,713 adolescents aged 11 to 15 years from 45 countries who participated in the 2018 Health Behaviour in School-aged Children (HBSC) study. Lifestyle behaviours, including physical inactivity, inadequate fruit and vegetable consumption, frequent soft drink consumption, alcohol use, and smoking, were used to examine the clustering of unhealthy behaviours. Multilevel mixed-effects logistic regression was employed to assess the associations between unhealthy behaviour clustering (≥3 unhealthy behaviours) and contextual factors at the individual, family, and school levels. **Results:** A high prevalence of clustered unhealthy behaviours was observed among adolescents, with 51.5% engaging in three or more unhealthy lifestyle behaviours. The odds increased with age (AOR: 1.79, 95% CI: 1.75, 1.84 for those aged ≥ 15 years), among males (AOR: 1.26, 95% CI: 123, 1.28), and among those experiencing higher academic pressure (AOR: 1.13, 95% CI: 1.09, 1.17 for very high academic pressure). In contrast, the odds were lower among adolescents from a higher family affluence background (AOR: 0.62 95% CI: 0.60, 0.65 for high), among adolescents living with both parents (AOR: 0.83, 95 CI: 0.81, 0.85), those reporting higher family support (AOR: 0.62, 95% CI: 0.60, 0.63 for high), higher peer support at school (AOR: 0.87, 95% CI: 0.84, 0.89 for high), and those reporting higher school satisfaction (AOR: 0.50, 95% CI: 0.48, 0.52 for very high). **Conclusions**: The study reveals that one in two adolescents engages in three or more unhealthy lifestyle behaviours. It emphasises the need to tackle this public health challenge through multisectoral interventions targeting individual-level and contextual factors at the family and school levels.

## 1. Introduction

The World Health Organization (WHO) defines adolescence as the period from 10 to 19 years, marked by rapid physical, cognitive, and socio-emotional changes that are essential for establishing a foundation for long-term health and wellbeing [1,2]. Unhealthy lifestyle behaviours, such as physical inactivity, poor diet, tobacco use, and excessive alcohol consumption, are often established during this period and usually persist into adulthood, increasing the risk of chronic diseases and premature mortality [2,3,4,5].

About 85% of adolescents worldwide do not meet the 60 min per day recommendation for physical activity, and 86% and 79% consume inadequate vegetables and fruits, respectively [6]. Over one in four adolescents currently drink alcohol, and six to eleven percent of teenagers reported smoking cigarettes at least once in the previous 30 days [7,8]. These individual unhealthy lifestyle habits usually occur in clusters, and about 82 to 92 percent of adolescents engage in two or more of these unhealthy lifestyle habits, which increases the risk of physical and mental health-related outcomes [9,10,11,12,13,14]. The clustering of these behaviours varies significantly by age, sex, family socioeconomic status (SES) and the level of social support received from family and friends [10,15,16,17,18,19,20,21].

In socioeconomically less developed nations, adolescents are more likely to adopt traditional lifestyles. These lifestyles include routines that are more physically active and diets rich in minimally processed foods. In contrast, more developed countries see a shift toward unhealthy habits due to increased access to processed foods and sedentary entertainment [22,23,24,25,26]. A recent global study found that adolescents in countries with higher levels of socioeconomic development were more likely to engage in clusters of unhealthy behaviours. These include physical inactivity, insufficient consumption of fruits and vegetables, and frequent intake of soft drinks and fast foods when compared to their peers in less developed nations [10].

Most existing cross-national studies on unhealthy lifestyle behaviours among adolescents are focused on low- and middle-income countries [6,9,10]. This focus may limit how we can apply the findings to high-income countries. Moreover, the impact of school-related factors such as peer support, feelings about school, academic pressure, and the socioeconomic status of families at school has not been examined. While theories like Social Cognitive Theory highlight the role of individuals and the environment in shaping behaviours, few studies take this approach fully [27]. Therefore, this study aims to explore the clustering of unhealthy lifestyle behaviours and its contextual factors at the individual, family, and school levels among adolescents in 45 countries across Europe, Central Asia, and North America.

## 2. Materials and Methods

### 2.1. Data Source and Study Population

This study utilised data from the 2018 Health Behaviour in School-aged Children (HBSC) survey. The HBSC survey is a collaborative, cross-national study conducted in countries across Europe, Central Asia, and North America, in partnership with the World Health Organization (WHO) Regional Office for Europe. The study population consists of adolescents attending school at ages 11, 13, and 15 years, with a recommended sample size of 1500 students for each age group, obtained through a two-stage cluster sampling method within each participating country [28].

Data were collected through self-reported questionnaires administered in the classroom. The survey utilises standard questionnaires to collect information across all participating countries, facilitating comparisons between countries and enabling national and cross-national data analysis. The questionnaires include mandatory questions that all countries must answer to create the cross-national dataset. Additionally, countries can select from optional question packages on specific topics and country-specific questions addressing issues of national significance. The source language for all items is English, with translations into the national languages. The cross-national data were processed and harmonised centrally by the HBSC Data Management Centre. A detailed description of this school-based survey is available elsewhere [28].

The dataset included 244,097 adolescents from 47 countries. However, two countries, Turkey and Greenland, were excluded because there were no data on alcohol consumption and/or smoking. Of the remaining 45 countries, with a total sample of 237,006 adolescents, 12% were excluded due to missing data for one or more variables: physical activity, fruit, vegetables, soft drinks, alcohol, and smoking.

Finally, a total of 210,713 adolescents with complete outcome variables from 45 countries were included in this study (Figure S1).

The sample sizes in each participating country ranged from 1367 in Norway to 14,332 in Wales, with a nearly equal distribution of both genders within each country (Table S1).

### 2.2. Study Outcome

This study included six lifestyle behaviours: physical activity, fruit and vegetable consumption, soft drink intake, alcohol use, and smoking.

Physical activity was measured using the question: “Over the past 7 days, on how many days were you physically active for a total of at least 60 min per day?” with response options of 0 days, 1 day, 2 days, 3 days, 4 days, 5 days, 6 days, or 7 days. We dichotomised physical activity into those who met the World Health Organization (WHO) recommendations for engaging in physical activity of at least 60 min per day and those who did not [29].

Fruits, vegetables, and soft drink consumption were assessed using the Food Frequency Questionnaire (FFQ) and categorised into daily consumers (once or more per day) and non-daily consumers (less than once a day) [30].

Alcohol consumption and cigarette smoking were evaluated through the following questions: “How many days have you consumed alcohol in the last 30 days?” and “How many days have you smoked cigarettes in the last 30 days?” The response options were as follows: “never,” “1–2 days,” “3–5 days,” “6–9 days,” “10–19 days,” “20–29 days,” and “30 days or more.” Both alcohol consumption and cigarette smoking were categorised into two groups: those who had never used in the past 30 days and those who had used at least once in the past 30 days preceding the survey [6]. A composite score reflecting unhealthy lifestyle behaviours was calculated by adding all individual unhealthy behaviours. Each unhealthy behaviour was given a score of “1”. In contrast, each healthy behaviour received a score of “0”, resulting in a total score ranging from 0 to 6, with “0” indicating the absence of all unhealthy behaviours and “6” indicating the presence of all unhealthy behaviours. The Supplementary File describes the definitions and coding (Table S2).

### 2.3. Contextual Factors

Eight individual/family level variables (age, sex, family affluence, living with parents, perceived family support, perceived peer support, school satisfaction, and school pressure), one aggregated school-level variable (average school-level family affluence), and one country-level variable (the geographical area of the study) were included in the analysis (Table S1).

Family wealth was measured using the six-item Family Affluence Scale (FAS). The six items were car ownership (0, 1, 2, or more), own bedroom (no = 0, yes = 1), computer ownership (0, 1, 2, 3, or more), number of bathrooms (0, 1, 2, 3, or more), dishwasher (no = 0, yes = 1), family holiday travel abroad during the last 12 months (0, 1, 2, 3, or more). A FAS score was calculated by adding the scores of the six items, ranging from 0 to 13. The summed score is transformed into a regression-based indicator for socioeconomic inequality using the ridit transformation [31]. The ridit transformation of the FAS score was performed by age group, gender, and country, and incorporating survey sample weights [32]. The ridit score was finally used to classify adolescents into the lowest 20% (low family affluence), middle 60% (medium family affluence), and highest 20% (high family affluence).

Perceived social support from family and peers was measured using questions adapted from the Multidimensional Scale of Perceived Social Support (MSPSS) [33]. Four questions were asked to assess adolescents’ perception of the social support they receive from their parents and peers. The response options ranged from (“very strongly disagree” = 1 to “very strongly agree” = 7). The total score for the four questions is calculated, ranging from 1 to 28. The scales demonstrate high internal consistency with a Cronbach’s α of 0.94 for perceived family support and 0.92 for perceived peer support. Finally, the perceived support levels from family and peers were divided into three categories using tertiles (low, medium, and high) to facilitate interpretation and comparison with the existing literature. Additionally, we repeated the analyses by fitting the continuous family and peer support scores in the multilevel model with restricted cubic splines (RCSs) with 3 knots placed at 10th, 50th, and 90th percentiles. We used the median values of the respective support scores as a reference to compare the odds of the outcome across the lower and higher support scores. *p*-values for non-linearity were tested by setting the coefficient of regression for the second splines equal to zero.

School satisfaction was measured using a single item measuring adolescents’ emotional and psychological attachment to school. The question asks: “How do you feel about school at present?” The responses were recoded into (“I don’t like it at all” = 0 to “I like it a lot” = 4).

School pressure was measured using a single item to assess the overall feeling of being pressured by academic work, thereby measuring school-related stress. The question asks: “How pressured do you feel by the schoolwork you have to do?” The responses were coded into (“Not at all” = 1 to “A lot” = 4).

The mean FAS score for adolescents within the same school was used to measure average family affluence at the school level [34].

The countries were geographically divided into Western, Eastern, Northern, and Southern European countries.

### 2.4. Statistical Analysis

#### 2.4.1. Data Preparation and Descriptive Analyses of Clusters of Lifestyle Behaviours

Data cleaning and processing were performed using R version 4.4.0 [35] and STATA/SE version 18 [36]. The weighted descriptive estimates were computed for all countries and compared across age categories, sex, and levels of family affluence. Mixed-effects linear regression was used to assess the statistical significance of mean differences across these categories. The clustering patterns of unhealthy lifestyle behaviours were summarised descriptively using the “upsetplot” function from STATA [37]. Using these functions, intersections between different sets of behaviours were plotted to explore how multiple unhealthy behaviours cluster within an individual.

#### 2.4.2. Multilevel Logistic Regression Analyses

Clustering of three or more unhealthy lifestyle behaviours was used as the primary outcome in this study. We have also conducted sensitivity analyses using different thresholds (two or more and four or more) to assess the robustness of the findings.

A multilevel mixed-effects logistic regression analysis was conducted with the sample weight specified in the model. This model accommodates the hierarchical data structure, where adolescents at level 1 are nested within schools at level 2, and schools are nested within countries at level 3.

Three models incorporating individual, school, and country-level variables were fitted. First, the null model, the empty, and intercept-only model without any covariates, was fitted to provide a baseline comparison with more complex models. The models we sequentially fitted with the final model incorporating individual, family, school and country-level covariates. Additionally, gender stratified multilevel models were conducted to compare the consistency of the findings across the subgroups.

The fixed effects were reported as adjusted odds ratios (AORs) and 95% confidence intervals (CIs). Random effects (measures of variation) at the school and country levels were presented in terms of intraclass correlation (ICC) and percentage change in variance (PCV) [38]. Multicollinearity between covariates included in the models was assessed using the Variance Inflation Factor (VIF). The mean VIF in the final reported model was 1.2, which is less than the recommended cut-off values of less than 5–10 [39].

Model fit was assessed using Akaike information criterion (AIC), deviance, and log-likelihood (LL) statistics [40]. A two-tailed *p*-value < 0.05 was considered to declare statistical significance.

## 3. Results

### 3.1. Prevalence of Individual Unhealthy Lifestyle Behaviours

**Figure 1:**
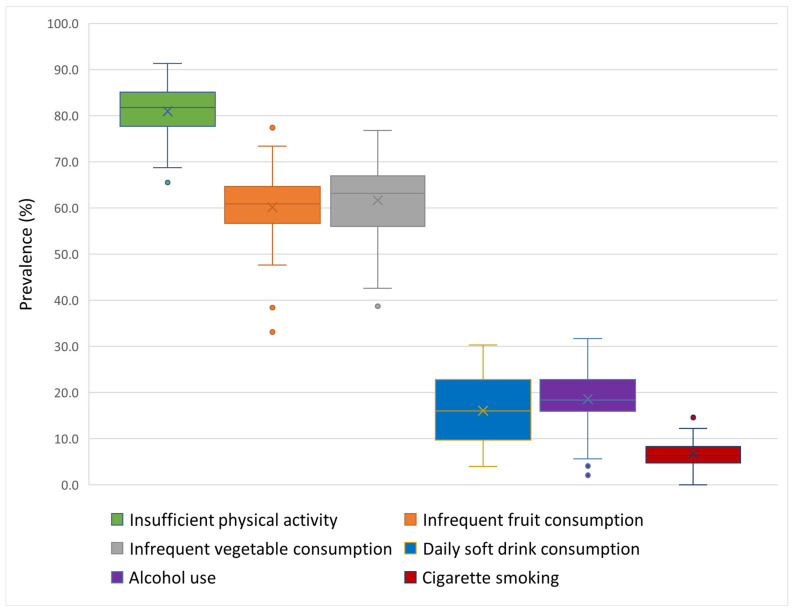
The boxplot shows the prevalence of unhealthy lifestyle behaviours among adolescents in 45 countries. The box represents the interquartile range (IQR), which contains the middle 50% of the data. Whiskers extend from the box to the minimum and maximum values. The symbol “×” denotes the mean values, while “●” represents the lowest or highest observed prevalence.

Across all countries, the overall prevalence of insufficient physical activity (less than 60 min per day) was 81%. Ranging from 65.5% in Kazakhstan to 91.3% in Italy. In addition, on average, 60% of adolescents across all countries consume fruit less frequently (<1 time per day), with national prevalences varying from 33.1% in Albania to 77.4% in Finland. Similarly, the prevalence of infrequent vegetable consumption (<1 time per day) ranged from 38.7% in Belgium (Flemish) to 76.8% in Turkey, averaging 61% across all countries. Daily soft drink consumption (≥1 time per day) varies widely across countries, ranging from 4% in Iceland to 30% in Belgium (Flemish), with an average of 15.7% across all countries. Likewise, alcohol consumption (≥1 time in the last 30 days) varies from 2.1% in Kazakhstan to 31.7% in Bulgaria, with an average of 19.3% across all countries. On average, 6.9% of adolescents smoke cigarettes (≥1 time in the last 30 days) across all countries, with national figures ranging from 3% in Iceland and Azerbaijan to 17.4% in Bulgaria (Table S3, Figure 1).

### 3.2. Clustering of Unhealthy Lifestyle Behaviours

The mean number of unhealthy behaviours slightly varies across countries, from 1.94 in Canada to 2.75 in Italy and Hungary. The number of unhealthy behaviours was significantly higher among adolescents aged 15 years and older compared to those under 15 years in all but three countries. A significant gender difference was observed in all but nine countries, with lower rates among females than among males. Additionally, in most countries, adolescents from affluent families engaged in a lower number of unhealthy behaviours than those from less affluent families (Table 1).

On average, 18.9% of adolescents had only one of the six unhealthy behaviours studied. The prevalence rates for co-occurring unhealthy behaviours are as follows: 24.3% for two behaviours, 35.2% for three behaviours, 12.0% for four behaviours, 3.7% for five behaviours, and 0.6% for six behaviours. There are considerable variations across countries and subregions (Table S4, Figure S2).

**Figure 2:**
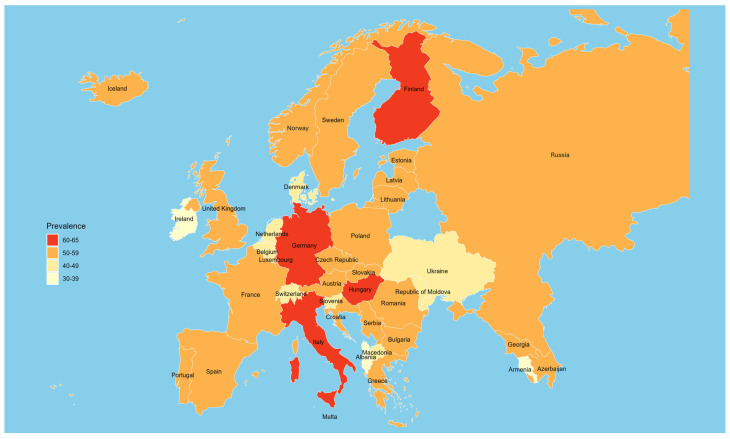
Prevalence of three or more unhealthy lifestyle behaviours by country, HBSC 2018.

The prevalence of clustered unhealthy behaviours was 75.8% for two or more behaviours, 51.5% for three or more, and 16.3% for four or more (Figure S3). The prevalence of three or more unhealthy behaviours varies widely across countries, with relatively higher prevalences observed in Finland, Germany, Hungary, and Italy, while the lowest were observed in Ireland, Albania, and Armenia (Figure 2, Table S5).

**Figure 3:**
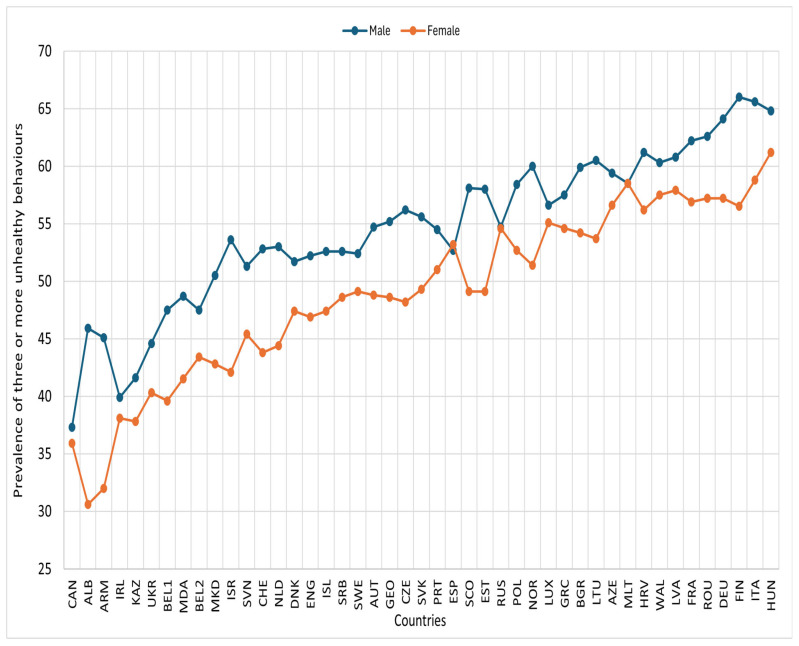
Prevalence of three or more unhealthy lifestyle behaviours by gender and country, HBSC 2018. Footnotes: ALB: Albania; ARM: Armenia; AUT: Austria; AZE: Azerbaijan; BEL1: Belgium (Flemish); BEL2: Belgium (French); BGR: Bulgaria; CAN: Canada; HRV: Croatia; CZE: Czech Republic; DNK: Denmark; ENG: England; EST: Estonia; FIN: Finland; FRA: France; GEO: Georgia; DEU: Germany; GRC: Greece; HUN: Hungary; ISL: Iceland; IRL: Ireland; ISR: Israel; ITA: Italy; KAZ: Kazakhstan; LVA: Latvia; LTU: Lithuania; LUX: Luxembourg; MKD: Macedonia; MLT: Malta; NLD: Netherlands; NOR: Norway; POL: Poland; PRT: Portugal; MDA: Republic of Moldova; ROU: Romania; RUS: Russia; SCO: Scotland; SRB: Serbia; SVK: Slovakia; SVN: Slovenia; ESP: Spain; SWE: Sweden; CHE: Switzerland; UKR: Ukraine; WAL: Wales.

**Figure 4:**
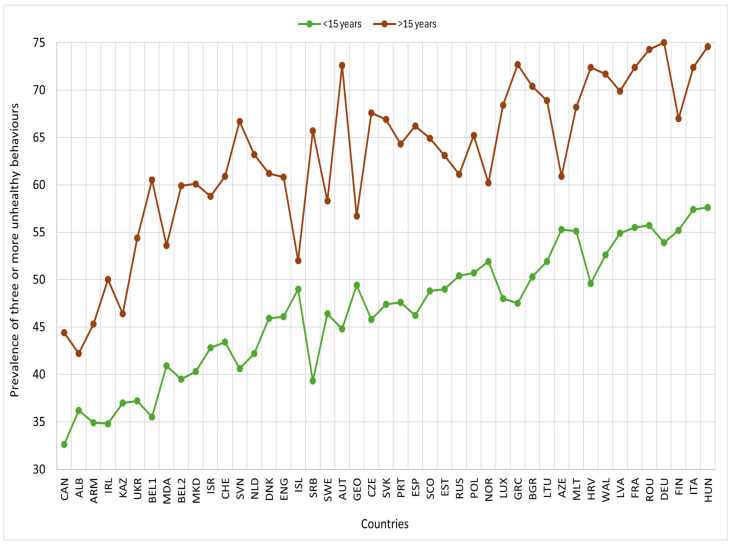
Prevalence of three or more unhealthy lifestyle behaviours by age category and country, HBSC 2018. Footnotes: ALB: Albania; ARM: Armenia; AUT: Austria; AZE: Azerbaijan; BEL1: Belgium (Flemish); BEL2: Belgium (French); BGR: Bulgaria; CAN: Canada; HRV: Croatia; CZE: Czech Republic; DNK: Denmark; ENG: England; EST: Estonia; FIN: Finland; FRA: France; GEO: Georgia; DEU: Germany; GRC: Greece; HUN: Hungary; ISL: Iceland; IRL: Ireland; ISR: Israel; ITA: Italy; KAZ: Kazakhstan; LVA: Latvia; LTU: Lithuania; LUX: Luxembourg; MKD: Macedonia; MLT: Malta; NLD: Netherlands; NOR: Norway; POL: Poland; PRT: Portugal; MDA: Republic of Moldova; ROU: Romania; RUS: Russia; SCO: Scotland; SRB: Serbia; SVK: Slovakia; SVN: Slovenia; ESP: Spain; SWE: Sweden; CHE: Switzerland; UKR: Ukraine; WAL: Wales.

**Figure 5:**
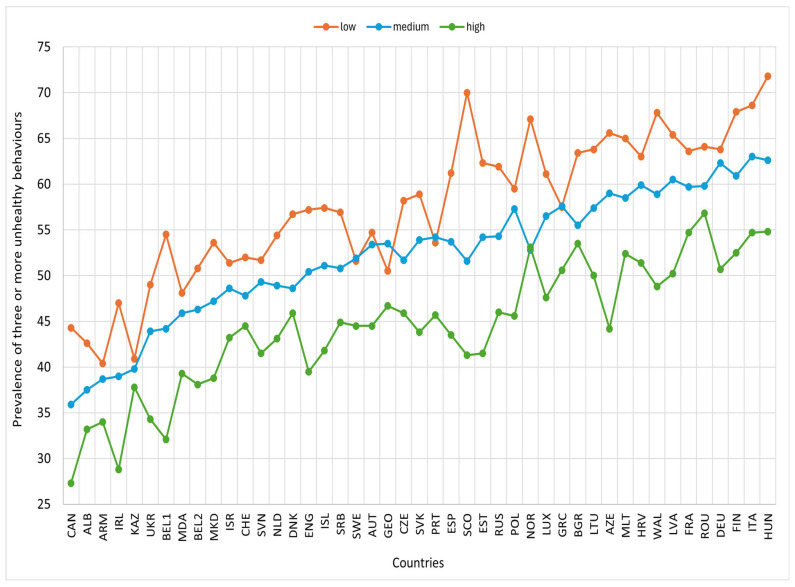
Prevalence of three or more unhealthy lifestyle behaviours by Family Affluence Scale (FAS) and country, HBSC 2018. Footnotes: ALB: Albania; ARM: Armenia; AUT: Austria; AZE: Azerbaijan; BEL1: Belgium (Flemish); BEL2: Belgium (French); BGR: Bulgaria; CAN: Canada; HRV: Croatia; CZE: Czech Republic; DNK: Denmark; ENG: England; EST: Estonia; FIN: Finland; FRA: France; GEO: Georgia; DEU: Germany; GRC: Greece; HUN: Hungary; ISL: Iceland; IRL: Ireland; ISR: Israel; ITA: Italy; KAZ: Kazakhstan; LVA: Latvia; LTU: Lithuania; LUX: Luxembourg; MKD: Macedonia; MLT: Malta; NLD: Netherlands; NOR: Norway; POL: Poland; PRT: Portugal; MDA: Republic of Moldova; ROU: Romania; RUS: Russia; SCO: Scotland; SRB: Serbia; SVK: Slovakia; SVN: Slovenia; ESP: Spain; SWE: Sweden; CHE: Switzerland; UKR: Ukraine; WAL: Wales.

In most countries, the prevalence of three or more unhealthy behaviours was higher among males than females. Similarly, the prevalences were higher among 15-year-olds and older compared to those under 15, and lower among adolescents with high or medium family affluence than those with low family affluence (Figure 3, Figure 4 and Figure 5).

The most common co-occurring combination of unhealthy behaviours among adolescents was insufficient physical activity and infrequent consumption of fruits and vegetables (Figure S4).

### 3.3. Association Between Multilevel Contextual Factors and Clustering of Unhealthy Behaviours

#### 3.3.1. Multilevel (Random Effects) Results

The baseline model, without predictor variables, reveals significant variance in the outcome variables: τ^2^ = 0.09 (*p* < 0.001) at the country level and τ^2^ = 0.23 (*p* < 0.0001) at the school level. The intraclass correlation coefficient (ICC) indicated that 2.5% of the variance in the outcome was attributable to differences between countries, while 8.9% was due to differences between schools. In the final model, which adjusted for individual, family, school, and country-level variables, the ICC was reduced to 2% at the country level and 5.7% at the school level. The explained variance in the final model was 21.4% at the country level and 43% at the school level. We compared the models using the AIC and log-likelihood (Table S6).

#### 3.3.2. Fixed Effects Analysis Results

**Figure 6:**
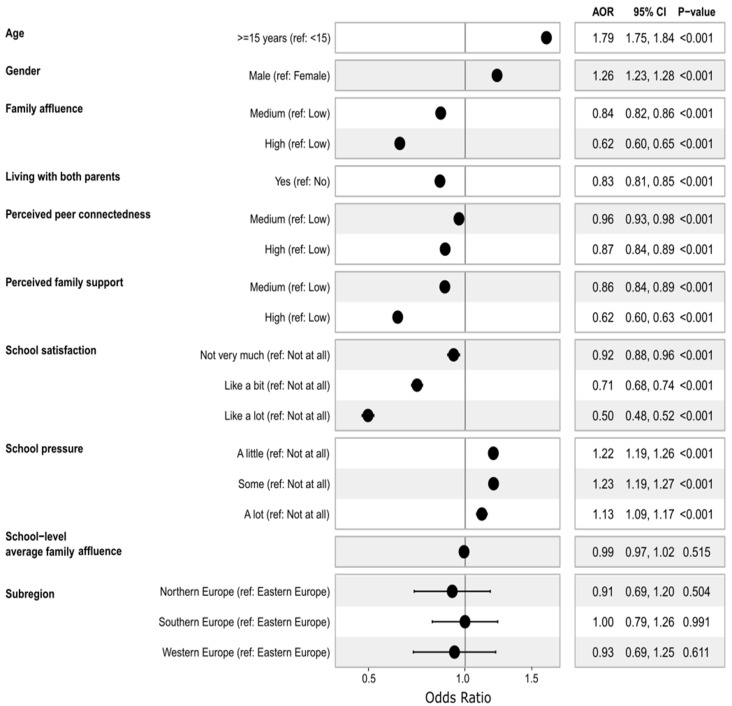
Mixed-effects logistic regression analysis of factors associated with three or more unhealthy behaviours among adolescents using the 2018 HBSC data. The model is mutually adjusted for all reported covariates and the Human Development Index (HDI). AOR, adjusted odds ratio.

Figure 6 presents the individual, school, and country-level factors associated with three or more unhealthy lifestyle behaviours from a multilevel mixed-effects logistic regression analysis. The factors associated with clustering three or more unhealthy lifestyle behaviours included age, gender, family affluence (FAS), living with parents, perceived family support, perceived peer support, school satisfaction, and school pressure.

Adolescents aged 15 and older had 79% higher odds of exhibiting clustering of three or more unhealthy behaviours than those under 15 (AOR = 1.79, 95% CI: 1.75, 1.84). Males had 26% higher odds of the outcome compared to their female counterparts (AOR = 1.26, 95% CI: 01.23, 1.28). Adolescents from affluent families had lower odds of engaging in multiple unhealthy behaviours than those from less affluent backgrounds. Those from high FAS had 38% lower odds (AOR = 0.62, 95% CI: 0.60, 0.65), while those in the medium FAS had 16% lower odds (AOR = 0.84, 95% CI: 0.82, 0.86) compared to the low FAS group.

Adolescents living with both parents had 17% reduced odds of engaging in multiple unhealthy behaviours compared to those not living with both parents (AOR = 0.83, 95% CI: 0.81, 0.85). Compared to those with low family support, those with medium and high family support had 14% (AOR = 0.86, 95% CI: 0.84, 0.89) and 38% (AOR = 0.60, 95% CI: 0.60, 0.63) lower odds, respectively. Similarly, compared to adolescents with low peer support, those with medium support had 4% reduced odds (AOR = 0.96, 95% CI: 0.93, 0.98), and those with high support had 13% lower odds (AOR = 0.87, 95% CI: 0.84, 0.89). Additionally, the non-linear association with peer and family support were further explored with a restricted cubic spline (RCS). Both family and peer support demonstrate a non-linear association with the odds of engaging in three or more unhealthy behaviours (*p*-non-linearity < 0.001). Compared to adolescents with a median peer support score, those with very low and very high support scores had lower odds of the outcome, while those with moderate scores demonstrated higher odds of the outcome. In contrast, adolescents with lower family support demonstrated higher odds of engaging in three or more unhealthy behaviours compared to those with median scores, and the odds dropped to less than one for those with higher family support above the median score (Figures S5 and S6).

Adolescents who reported higher school satisfaction had significantly lower odds of engaging in three or more unhealthy behaviours, with adjusted odds ratios of 0.92 (95% CI: 0.88, 0.96), 0.71 (95% CI: 0.68, 0.74), and 0.50 (95% CI: 0.48, 0.52) for moderate, high, and very high levels of school satisfaction, respectively, compared to those with lower school satisfaction scores.

In contrast, higher levels of perceived academic pressure from the school were associated with increased odds of engaging in three or more unhealthy behaviours, with adjusted odds ratios of 1.22 (95% CI: 1.19, 1.26), 1.23 (95% CI: 1.19, 1.27), and 1.13 (95% CI: 1.09, 1.17) for moderate, high, and very high academic pressure, respectively, compared to those with a lower academic pressure score.

Additional sensitivity analyses were also conducted with different cut-off values in the overall and gender-stratified sample, using two or more, and four or more unhealthy behaviour clusters. The findings remain consistent, except that higher peer support is associated with higher odds of engaging in four or more unhealthy behaviours (Tables S7 and S8).

## 4. Discussion

This study examined the clustering of six unhealthy lifestyle behaviours and its contextual determinants among adolescents in 45 nations in Europe, Central Asia, and North America. The behaviours include insufficient physical activity, inadequate fruit and vegetable intake, frequent soft drink consumption, alcohol use, and cigarette smoking. The three most common unhealthy behaviours identified include insufficient physical activity (81%) and low fruit and vegetable intake, over 60% for both. These finding aligns with previous global and regional studies reporting a high prevalence [9,10].

This study shows that 75.8% of adolescents engage in at least two unhealthy behaviours, 51.5% in three or more, and 16.3% in four or more. These findings are comparable but slightly higher in the prevalence of at least two unhealthy behaviours compared to a previous multicentre study in Africa, China, and India, which reported a prevalence of 90% for at least one unhealthy behaviour and 55% for two or more [41]. A similar high prevalence of clustered behaviours was reported in a previous global school-based study [9] and in the United States [17]. Given the high prevalence of co-occurring unhealthy behaviours among adolescents, interventions targeting multiple unhealthy behaviours might help avert this trend. For instance, a study examining the effectiveness of a school-based interventions found that targeting energy balance behaviours, including increasing physical activity, fruit and vegetable consumption, and limiting sedentary time, led to greater reductions in smoking and alcohol use than an intervention that was solely focused on substance-use prevention [42]. A similar synergistic effect was observed where addressing multiple behaviours yields a stronger result [43]. Despite these advantages, interventions that target multiple behaviours have limitations, such as complexity in implementation, variation in effectiveness across targeted behaviours, and difficulty in maintaining positive behavioural changes over time compared to the intervention targeting a specific unhealthy behaviour at a time [44,45]. Therefore, it is important to weigh the benefits and drawbacks of both approaches in developing adolescent health interventions.

In this study, we found that adolescents aged 15 years or older had 79% higher odds of exhibiting clustering of three or more unhealthy behaviours compared to those under 15. This finding aligns with a previous study conducted using a global sample of adolescents [10] and studies conducted in the United States and Canada [17,46,47]. The findings highlight the importance of early health promotion initiatives, as the accumulation of unhealthy behaviours tends to increase with age.

This study also found that males had 26% higher odds of clustering of three or more unhealthy behaviours compared to their female counterparts. In general, conflicting results have been reported across studies regarding gender differences in the clustering of unhealthy behaviours, with some studies reporting higher odds among females, and others reporting the opposite [48,49,50]. This variation could be related to the number of individual unhealthy behaviours considered in the clustering analysis, which may contribute to these differences. For example, in this study, females had a higher prevalence of insufficient physical activity, while males exhibited higher prevalence rates of inadequate fruit and vegetable intake, frequent consumption of soft drinks, alcohol use, and cigarette smoking. Similar findings have been reported in previous studies [51]. These findings call for gender-specific interventions focusing on increasing physical activity among females and addressing poor diet and substance use among males.

Adolescents from higher socioeconomic status (SES) families were 16% to 38% less likely to exhibit clustered unhealthy behaviours than those from low socioeconomic status families. This result is supported by the findings of systematic reviews that reported adolescents from low SES backgrounds have poor dietary habits, lower levels of physical activity, and are more likely to smoke cigarettes than their counterparts with high SES backgrounds [52,53]. In addition, adolescents from a low SES may have limited access to healthy food options, since healthy foods are relatively more expensive than easily accessible alternatives such as fast foods [54]. Similarly, research indicates that adolescents from low SES backgrounds are more likely to smoke and use alcohol than their peers from higher SES backgrounds [55]. Regarding physical activity, those from low SES backgrounds face several challenges, such as limited access to safe recreational spaces, financial issues, lack of parental support, and poor health literacy, leading to low physical activity participation compared to their counterparts from higher SES backgrounds [56,57,58].

This study also found that adolescents living with both parents had reduced odds of clustered unhealthy behaviours. Living with both parents has been reported as a protective factor for health-related behaviours, as adolescents living with both parents were less likely to report low life satisfaction, cigarette smoking, and episodes of drunkenness compared to those living with a single parent or in a stepfamily [59]. Adolescents living with both parents often engage in more health-promoting behaviours, such as better stress management, healthier diets, stronger social support, greater appreciation for life, increased health responsibility, and regular physical activity. In contrast, adolescents living with a single parent tend to score lower across various health behaviours domains, which may be due to reduced supervision, limited resources, and increased stress [60,61,62]. In this study, having supportive parents is associated with reduced engagement in risk behaviours, such as smoking, drinking, or physical inactivity, consistent with a previous study [63]. This finding is also supported in theory by the ecological model of health behaviour, which describes parental support as the emotional, instrumental, informational, and modelling support given by parents that affects a child’s or adolescent’s development, choices, and behaviours [64,65].

This study found that adolescents with high peer support at school and those experiencing high school satisfaction had lower odds of clustered unhealthy behaviours. On the other hand, those with high academic pressure had higher odds of clustered unhealthy behaviours. These findings highlight the importance of the school environment in shaping adolescent lifestyle behaviour. Enhancing positive peer engagement, improving student satisfaction with the school environment, and minimising undue academic pressure could contribute to reducing the odds of clustered unhealthy behaviours [66,67,68].

### Strengths and Limitations

Among the strengths, this study utilised a large nationally representative dataset from the Health Behaviour in School-aged Children (HBSC) survey, which uses consistent methodology across countries, making cross-national comparisons valid. In addition, the study employed multilevel logistic regression to adjust for within- and between-country variations, which enhances the robustness of the study’s findings. We also conducted multiple sensitivity analyses using alternative thresholds for clustering of unhealthy behaviours (≥2 and ≥4) in addition to the primary outcome (≥3) reported in this study. The sensitivity analyses confirmed consistent results across the thresholds, indicating the robustness of the findings.

However, the study’s findings should be interpreted in light of the following limitations that need to be noted. First, the survey response rates at the school and student levels may vary across countries, which may limit the generalizability of the findings to adolescents within each country and European countries in general. Second, as most countries in this study are European, the findings of this study might not be generalisable to other regions with different socio-economic settings. Third, the lifestyle behaviours examined in this study were self-reported, which may be subject to recall and social desirability biases, resulting in a conservative estimation of prevalences. For instance, dietary behaviours were measured using a food frequency questionnaire (FFQ), which may result in an over- or underestimation of the prevalence. Nevertheless, FFQs are generally effective in capturing dietary patterns and are suitable for use in large-scale surveys [69]. In addition to alcohol and tobacco use, the Health Behaviour in School-aged Children (HBSC) survey collected cannabis use; due to missing records for most of the surveyed countries, the data were excluded from the current study. This study was conducted using a data from a school survey, which may have excluded adolescents that were out of school. However, most European countries have high enrolment in primary and lower-secondary education. Finally, due to the cross-sectional design, this study is unable to capture seasonal variation in the reported behaviours or establish a causal association between the behaviours and explanatory variables.

## 5. Conclusions

This study revealed a high prevalence of clustered unhealthy lifestyle behaviours, which include physical inactivity and infrequent consumption of fruits and vegetables, soft drink consumption, alcohol use, and cigarette smoking among adolescents in 45 countries across Europe, Central Asia, and North America. More than three in four adolescents exhibit at least two unhealthy behaviours, and 51.5% exhibit three or more. The current study findings demonstrate that multilevel contextual factors at the individual level (age and gender), family level (family structure, socioeconomic status, and support) and school level (perceived peer support, school satisfaction, and academic pressure) are significantly associated with the clustering of these unhealthy behaviours. These results reinforce the need to address the clustering of unhealthy behaviours among school-aged adolescents holistically, considering multilevel contextual factors.

## Figures and Tables

**Table 1:** Mean number of unhealthy behaviours among adolescents by age, sex, and family affluence among adolescents using the 2018 HBSC data.

Countries	Overall Mean (SD)	Age		Sex		Family Affluence (FAS)		
		<15 Years	>=15 Years	Male	Female	Low	Medium	High
Non-European								
Canada	1.94 (1.2)	1.79	2.26 ***	1.95	1.94 *	2.17	1.93 ***	1.66 ***
Israel	2.37 (1.2)	2.22	2.69 ***	2.50	2.24 ***	2.53	2.39	2.21 *
Kazakhstan	2.06 (1.1)	2.00	2.21 ***	2.11	2.01 **	2.12	2.07	1.99 **
Eastern Europe								
Armenia	2.16 (1.1)	2.04	2.41 ***	2.35	1.99 ***	2.21	2.18	2.04 **
Azerbaijan	2.41 (1.0)	2.36	2.46	2.48	2.34 **	2.54	2.42 *	2.20 ***
Bulgaria	2.72 (1.3)	2.49	3.20 ***	2.76	2.69	2.94	2.68 ***	2.63 ***
Czech Republic	2.46 (1.3)	2.22	3.02 ***	2.55	2.37 ***	2.60	2.45 ***	2.32 ***
Georgia	2.51 (1.1)	2.44	2.65	2.62	2.40 ***	2.51	2.54	2.39
Hungary	2.75 (1.3)	2.53	3.27 ***	2.80	2.72 *	2.96	2.75 **	2.56 **
Poland	2.51 (1.2)	2.33	2.88 ***	2.58	2.45 ***	2.60	2.57	2.23 ***
Republic of Moldova	2.26 (1.1)	2.13	2.52 ***	2.38	2.14 ***	2.31	2.28	2.13 **
Romania	2.69 (1.2)	2.55	3.17 ***	2.77	2.60 ***	2.82	2.67 **	2.60 **
Russia	2.39 (1.1)	2.26	2.59 ***	2.40	2.38	2.58	2.37 ***	2.18 ***
Slovakia	2.47 (1.3)	2.29	2.97 ***	2.56	2.37 ***	2.60	2.49	2.27 ***
Ukraine	2.17 (1.2)	2.01	2.55 ***	2.23	2.12 **	2.34	2.21 *	1.94 ***
Northern Europe								
Denmark	2.39 (1.2)	2.22	2.93 ***	2.44	2.34 **	2.58	2.37 ***	2.25 ***
England	2.34 (1.2)	2.21	2.75 ***	2.38	2.29	2.54	2.37 *	2.04 ***
Estonia	2.41 (1.2)	2.25	2.74 ***	2.51	2.31 ***	2.61	2.43 ***	2.11 ***
Finland	2.67 (1.2)	2.44	2.92 ***	2.80	2.55 ***	2.78	2.66 *	2.56
Iceland	2.18 (1.1)	2.14	2.27 ***	2.24	2.12 **	2.35	2.22 ***	1.95 ***
Ireland	2.04 (1.2)	1.89	2.42 ***	2.06	2.02 *	2.29	2.02 ***	1.77 ***
Latvia	2.63 (1.1)	2.46	3.05 ***	2.65	2.62	2.78	2.66 *	2.43 ***
Lithuania	2.55 (1.2)	2.36	2.98 ***	2.65	2.45 ***	2.71	2.56	2.37 ***
Norway	2.44 (1.1)	2.30	2.61	2.55	2.34 **	2.69	2.39 ***	2.40 *
Scotland	2.49 (1.2)	2.31	2.95 ***	2.61	2.39 ***	2.89	2.46 ***	2.17 ***
Sweden	2.39 (1.1)	2.24	2.65 ***	2.40	2.37	2.42	2.42	2.21 **
Wales	2.64 (1.2)	2.39	3.14 ***	2.66	2.62	2.88	2.63 ***	2.37 ***
Southern Europe								
Albania	2.12 (1.2)	2.06	2.38 *	2.33	1.95 ***	2.22	2.13	2.02
Croatia	2.68 (1.3)	2.36	3.17 ***	2.75	2.62 **	2.8	2.72	2.47 **
Greece	2.62 (1.2)	2.36	3.14 ***	2.67	2.58 *	2.66	2.67	2.47
Italy	2.75 (1.1)	2.56	3.17 ***	2.83	2.69 ***	2.92	2.77 *	2.57 ***
Macedonia	2.30 (1.3)	2.08	2.78 ***	2.42	2.19 ***	2.52	2.31 **	2.10 ***
Malta	2.67 (1.2)	2.53	3.09 ***	2.67	2.67	2.83	2.67 **	2.49 ***
Portugal	2.49 (1.1)	2.32	2.86 ***	2.52	2.46 *	2.51	2.52	2.33 ***
Serbia	2.46 (1.4)	2.06	3.01 ***	2.52	2.40	2.64	2.46 **	2.31 ***
Slovenia	2.32 (1.2)	2.07	2.91 ***	2.40	2.24 ***	2.39	2.34	2.16 ***
Spain	2.52 (1.2)	2.30	2.95 ***	2.50	2.54	2.73	2.52 **	2.33 ***
Western Europe								
Austria	2.46 (1.2)	2.23	3.17 ***	2.53	2.40 ***	2.52	2.51	2.28 **
Belgium (Flemish)	2.31 (1.2)	2.07	2.83 ***	2.40	2.22 ***	2.56	2.34 **	2.01 ***
Belgium (French)	2.36 (1.2)	2.17	2.82 ***	2.42	2.30 *	2.49	2.39 *	2.17 ***
France	2.68 (1.2)	2.53	3.15 ***	2.73	2.63 ***	2.73	2.69	2.58 **
Germany	2.67 (1.2)	2.43	3.20 ***	2.76	2.6 ***	2.68	2.74	2.44 *
Luxembourg	2.57 (1.2)	2.30	2.95 ***	2.61	2.53 **	2.70	2.59	2.36 **
Netherlands	2.43 (1.2)	2.23	2.87 ***	2.53	2.32 ***	2.57	2.42 *	2.30 **
Switzerland	2.38 (1.2)	2.20	2.86 ***	2.50	2.27 ***	2.46	2.38	2.26 ***

Footnotes: Unhealthy behaviours include insufficient physical activity, infrequent consumption of fruits, infrequent consumption of vegetables, frequent consumption of soft drinks, alcohol consumption, and cigarette smoking; SD, standard deviation. *** *p* < 0.0001, ** *p* < 0.01, and * *p* < 0.05. (*p*-values are from mixed-effects linear regression).

## Data Availability

This study utilised secondary data from the Health Behaviour in School-aged Children (HBSC) survey. The authors do not have the right to share the data. Access can be sought through the HBSC Data Management Centre at: https://hbsc.org/data/, accessed on 23 May 2024.

## Supplementary Materials

The following supporting information can be downloaded at: https://www.mdpi.com/article/10.3390/nu17213388/s1, Figure S1: Flow chart illustrating the sample selection process using the 2018 HBSC; Figure S2: Prevalence of one or more unhealthy behaviours among adolescents by European regions, HBSC, 2018; Figure S3: Prevalence of co-occurring unhealthy lifestyle behaviours among adolescents, HBSC, 2018; Figure S4: Clustering of unhealthy lifestyle behaviours among adolescents, HBSC, 2018; Figure S5: Non-linear association between family support and the odds of three or more unhealthy behaviours among adolescents. Odds ratios (dashed line) and 95% confidence intervals (upper and lower dashed lines) were estimated using restricted cubic spline functions with three knots from the fully adjusted model *. The reference value (OR = 1) corresponds to the median peer support score of 26. Grey bars represent the distribution of participants (right axis); Figure S6: Non-linear association between peer support and the odds of three or more unhealthy behaviours among adolescents. Odds ratios (dashed line) and 95% confidence intervals (upper and lower dashed lines) were estimated using restricted cubic spline functions with three knots from the fully adjusted model*. The reference value (OR = 1) corresponds to the median peer support score of 23. Grey bars represent the distribution of participants (right axis); Table S1: Characteristics of survey samples included in the study on the clustering of unhealthy lifestyle behaviours and contextual determinants in adolescents, HBSC, 2018; Table S2: Coding of variables used in the study on the clustering of unhealthy lifestyle behaviours and contextual determinants in adolescents, HBSC, 2018; Table S3: Prevalence of unhealthy behaviours among adolescents, HBSC, 2018; Table S4: Prevalence of clusters of unhealthy behaviours by country, HBSC, 2018; Table S5: Prevalence of three or more unhealthy behaviours by sex using the 2018 HBSC survey; Table S6: Model comparison statistics and random effect parameters for mixed-effects logistic regression analysis of factors associated with three or more unhealthy behaviours among adolescents using the 2018 HBSC data; Table S7: Mixed-effects logistic regression analysis of factors associated with two or more unhealthy behaviours among adolescents using the HBSC 2018 survey; Table S8: Mixed-effects logistic regression analysis of factors associated with four or more unhealthy behaviours among adolescents using the HBSC 2018 survey.
