# Supplementary material for: Clustering of Unhealthy Lifestyle Behaviours and Its Contextual Determinants in Adolescents: A Multilevel Analysis of School-Based Surveys in 45 Countries
Source: Nutrients. 2025 Oct 28;17(21):3388. doi: 10.3390/nu17213388 (PMC12610362; doi:10.3390/nu17213388)
Supplement: Supplementary file 1 [file nutrients-17-03388-s001.zip › nutrients-3880258-supplementary.pdf]

Clustering of Unhealthy Lifestyle Behaviours and Its Contextual Determinants in Adolescents: A Multilevel Analysis of School-Based Surveys in 45 Countries

Table S1: Characteristics of survey samples included in the study on the clustering of unhealthy lifestyle behaviours and contextual determinants in adolescents, HBSC, 2018.

Countries	Year of survey	Sample size	Male (%)	Female (%)
Albania	2017	1765	45.5	54.5
Armenia	2017	4717	49.9	50.1
Austria	2018	4129	49.3	50.7
Azerbaijan	2017	4586	48.2	51.8
Belgium (Flemish)	2018	4333	49.5	50.5
Belgium (French)	2018	5578	50.0	50.0
Bulgaria	2018	4548	48.4	51.6
Canada	2018	12950	47.5	52.5
Croatia	2018	5169	51.0	49.0
Czech Republic	2018	11564	50.1	49.9
Denmark	2018	3181	48.6	51.4
England	2018	3397	51.6	48.4
Estonia	2018	4725	50.1	49.9
Finland	2018	3146	49.3	50.7
France	2018	9170	49.2	50.8
Georgia	2018	4242	49.7	50.3
Germany	2018	4347	47.0	53.0
Greece	2018	3863	49.9	50.1
Greenland	2018	1243	47.9	52.1
Hungary	2018	3789	47.2	52.8
Iceland	2018	6996	50.2	49.8
Ireland	2018	3833	50.6	49.4

Israel	2018	7712	50.4	49.6
Italy	2018	4144	48.2	51.8
Kazakhstan	2017	4868	50.4	49.6
Latvia	2018	4412	49.6	50.4
Lithuania	2018	3797	50.4	49.6
Luxembourg	2018	4070	50.1	49.9
Macedonia	2018	4658	48.9	51.1
Malta	2018	2576	48.1	51.9
Netherlands	2017	4698	51.3	48.7
Norway	2018	3127	48.5	51.5
Poland	2018	5224	49.2	50.8
Portugal	2018	6126	47.8	52.2
Republic of Moldova	2018	4686	50.1	49.9
Romania	2018	4567	48.8	51.2
Russia	2018	4281	47.7	52.3
Scotland	2018	5021	48.2	51.8
Serbia	2018	3933	49.5	50.5
Slovakia	2018	4785	51.3	48.7
Slovenia	2018	5667	51.4	48.6
Spain	2018	4320	48.3	51.7
Sweden	2017	4185	49.7	50.3
Switzerland	2018	7510	50.4	49.6
Turkey	2019	5848	48.8	51.2
Ukraine	2018	6660	51.4	48.6
Wales	2017	15951	50.1	49.9
Total	2017/2018	244,097	49.5	50.5

Footnotes**:** Percentages in the table are weighted for the complex survey sample.

Table S2: Coding of variables used in the study on the clustering of unhealthy lifestyle behaviours and contextual determinants in adolescents, HBSC, 2018.

Variables	Survey questions	Coding
**Dependent variable**		
Physical activity	Over the past 7 days, on how many days were you physically active for a total of at least 60 minutes per day?	7 days (0)<br>0-6 days (1)
Fruit	How many times a week do you usually eat or drink fruits?	Once or more every day (0)<br>Less than once per day (1)
Vegetable	How many times a week do you usually eat or drink vegetables?	Once or more every day (0)<br>Less than once per day (1)
Soft drink	How many times a week do you usually eat or drink Coke or other soft drinks that contain sugar?	Less than once per day (0)<br>Once or more every day (1)
Alcohol	On how many days (if any) have you drunk alcohol in the last 30 days?	never (0)<br>At least once (1)
Cigarette	On how many days (if any) have you smoked cigarettes in the last 30 days?	never (0)<br>At least once (1)
**Individual-level variables**		
Age	Date of birth (MM/YYYY)	< 15 years (0)<br>≥ 15 years (1)
Sex	Are you a girl or a boy?	Male (0)<br>Female (1)
Family Affluence Scale (FAS)	1. Does your family own a car, van or truck?<br>2. Do you have your own bedroom for yourself?<br>3. How many computers does your family own (including laptops and tablets, not including game consoles and smartphones)?	Low family affluence (0)<br>Medium family affluence (1)<br>High family affluence (2)

	4. How many bathrooms (room with a bath/shower or both) are in your home?<br>5. Does your family have a dishwasher at home?<br>6. How many times did you and your family travel out of [insert country here] for a holiday/vacation last year?	
Living with parents	Please answer this first question for the home where you live all or most of the time and tick the people who live there.<br>1. Mother<br>2. Father<br>3. Stepmother (or father's girlfriend/partner)<br>4. Stepfather (or mother's boyfriend/partner)<br>5. I live in a foster home or children's home<br>6. Someone or somewhere else (e.g., siblings, grandparents)	Not living with both parents (0)<br>Living with both parents (1)
Perceived family support	We are interested in how you feel about the following statements. Please show how much you agree or disagree with each one.<br>1. My family really tries to help me<br>2. I get the emotional help and support I need from my family<br>3. I can talk about my problems with my family<br>4. My family is willing to help me make decisions<br>Response:<br>Very strongly disagree (1) to Very strongly agree (7)	Low (0)<br>Medium (1)<br>High (2)
Perceived peer support	We are interested in how you feel about the following statements. Please show how much you agree or disagree with each one.<br>1. My friends really try to help me<br>2. I can count on my friends when things go wrong<br>3. I have friends with whom I can share my joys and sorrows<br>4. I can talk about my problems with my friends<br>Response:	Low (0)<br>Medium (1)<br>High (2)

	Very strongly disagree (1) to Very strongly agree (7)	
School satisfaction	How do you feel about school at present?<br>1. I like it a lot<br>2. I like it a bit<br>3. I don't like it very much<br>4. I don't like it at all	I don't like it at all (0)<br>I don't like it very much (1)<br>I like it a bit (2)<br>I like it a lot (3)
School pressure	How pressured do you feel by the schoolwork you have to do?<br>1. Not at all<br>2. A little<br>3. Some<br>4. A lot	Not at all (0)<br>A little (1)<br>Some (2)<br>A lot (3)
**School-level variable**		
average school-level family affluence	The mean FAS score for adolescents within the same school was used to measure average family affluence at the school level.	Mean FAS Score (ratio variable)
**Country-level variable**		
Geographical region	1. Western Europe<br>2. Eastern<br>3. Northern<br>4. Southern	Western Europe (0)<br>Eastern (1)<br>Northern (2)<br>Southern (3)

Table S3: Prevalence of unhealthy behaviours among adolescents, HBSC, 2018.

Countries	Insufficient PA (<60 minutes /day)	Infrequent fruit consumption (<1 time/day)	Infrequent vegetable consumption (<1 time/day)	Daily soft drink consumption (≥1 time (s)/day)	Alcohol consumption in last 30 days (≥1 time (s)/30 days)	Cigarette smoking in last 30 days (≥1 time (s)/30 days)
Albania	79.3 (77.4,81.2)	33.1 (30.9,35.4)	52.5 (50.2,54.8)	27.2 (25.2,29.3)	15.1 (13.4,16.8)	6.7 (5.6,8.0)
Armenia	73.6 (72.3,74.9)	38.4 (37.0,39.8)	53.4 (52.0,54.8)	28.0 (26.7,29.3)	20.1 (18.9,21.3)	4.6 (4.0,5.2)
Austria	79.6 (78.3,80.8)	57.5 (56.0,59.0)	67.0 (65.6,68.4)	16.8 (15.7,18.0)	21.2 (19.9,22.5)	6.8 (6.0,7.6)
Azerbaijan	83.0 (81.8,84.0)	60.9 (59.5,62.3)	65.4 (64.0,66.8)	13.8 (12.9,14.9)	4.1 (3.4,4.9)	3.0 (2.4,3.8)
Belgium (Flemish)	81.3 (80.1,82.4)	60.8 (59.3,62.2)	38.7 (37.3,40.2)	23.4 (22.1,24.7)	22.2 (21.0,23.5)	4.2 (3.6,4.9)
Belgium (French)	83.4 (82.4,84.4)	52.6 (51.2,53.9)	42.6 (41.3,44.0)	30.3 (29.1,31.5)	21.3 (20.3,22.5)	5.4 (4.8,6.1)
Bulgaria	77.1 (75.8,78.3)	61.8 (60.4,63.2)	57.3 (55.9,58.8)	26.6 (25.4,28.0)	31.7 (30.4,33.1)	17.4 (16.3,18.5)
Canada	73.9 (72.8,75.1)	47.6 (46.3,48.9)	47.0 (45.7,48.3)	5.0 (4.5,5.6)	17.9 (16.9,18.9)	4.0 (3.5,4.5)
Croatia	77.3 (76.2,78.5)	65.5 (64.2,66.8)	72.6 (71.3,73.8)	16.4 (15.4,17.4)	25.3 (24.1,26.5)	12.2 (11.3,13.1)
Czech Republic	81.9 (81.2,82.7)	54.1 (53.1,55.1)	63.2 (62.3,64.2)	13.8 (13.1,14.4)	24.9 (24.1,25.8)	8.3 (7.8,8.9)
Denmark	89.4 (88.3,90.4)	62.0 (60.2,63.6)	53.5 (51.8,55.3)	6.2 (5.4,7.1)	21.8 (20.4,23.3)	5.4 (4.6,6.2)
England	84.6 (83.3,85.8)	59.3 (57.6,61.1)	56.4 (54.6,58.2)	9.8 (8.7,11.0)	20.3 (18.8,21.9)	4.8 (4.1,5.6)
Estonia	84.5 (83.4,85.5)	58.6 (57.2,60.0)	67.5 (66.2,68.8)	5.6 (4.9,6.2)	15.7 (14.6,16.8)	9.1 (8.3,9.9)
Finland	69.9 (68.2,71.6)	77.4 (75.8,78.9)	69.7 (68.0,71.4)	4.1 (3.4,4.9)	25.0 (23.0,27.0)	8.4 (7.5,9.5)
France	89.5 (88.8,90.2)	64.7 (63.6,65.9)	61.5 (60.4,62.7)	22.8 (21.8,23.8)	22.8 (21.8,23.9)	6.8 (6.2,7.4)
Georgia	78.8 (77.5,80.0)	55.3 (53.8,56.8)	63.2 (61.7,64.6)	27.5 (26.1,28.9)	21.5 (20.3,22.8)	5.2 (4.6,5.9)
Germany	87.1 (86.1,88.1)	61.8 (60.3,63.2)	72.2 (70.8,73.5)	13.9 (12.9,14.9)	25.1 (23.8,26.5)	7.0 (6.3,7.8)
Greece	83.6 (82.4,84.7)	68.9 (67.4,70.3)	66.6 (65.1,68.1)	5.9 (5.2,6.7)	29.8 (28.3,31.2)	7.9 (7.1,8.8)
Greenland	79.5 (77.1,81.7)	60.8 (57.9,63.6)	62.5 (59.7,65.2)	25.2 (22.8,27.8)	6.4 (5.1,8.0)	N/A
Hungary	80.3 (79.0,81.6)	67.2 (65.7,68.7)	71.7 (70.2,73.1)	24.4 (23.1,25.8)	25.5 (24.1,26.9)	9.8 (8.7,10.9)
Iceland	79.5 (78.5,80.4)	60.0 (58.9,61.2)	65.8 (64.7,66.9)	4.0 (3.6,4.5)	5.6 (5.1,6.2)	3.0 (2.6,3.4)
Ireland	73.6 (72.2,75.0)	56.6 (55.0,58.1)	55.8 (54.2,57.4)	5.7 (5.0,6.5)	9.0 (8.1,9.9)	3.8 (3.2,4.4)
Israel	88.0 (87.2,88.8)	53.6 (52.2,55.0)	46.0 (44.5,47.4)	25.4 (24.2,26.7)	9.8 (9.0,10.6)	6.6 (5.9,7.4)
Italy	91.3 (90.4,92.1)	64.0 (62.5,65.4)	72.9 (71.5,74.2)	12.6 (11.6,13.6)	23.8 (22.5,25.1)	11.5 (10.6,12.5)

Kazakhstan	65.5 (64.1,66.9)	62.2 (60.8,63.6)	57.1 (55.7,58.5)	16.6 (15.5,17.7)	2.1 (1.8,2.6)	3.1 (2.6,3.6)
Latvia	81.2 (80.0,82.3)	73.2 (71.9,74.5)	72.8 (71.4,74.1)	6.2 (5.5,7.0)	20.5 (19.3,21.7)	10.2 (9.3,11.1)
Lithuania	81.7 (80.5,82.9)	64.7 (63.2,66.3)	65.8 (64.2,67.3)	12.6 (11.6,13.7)	16.6 (15.4,17.8)	14.6 (13.5,15.8)
Luxembourg	86.0 (84.9,87.1)	61.6 (60.1,63.1)	62.8 (61.3,64.3)	22.8 (21.5,24.1)	16.4 (15.3,17.6)	8.1 (7.3,9.0)
Macedonia	68.7 (67.4,70.1)	55.6 (54.1,57.0)	54.0 (52.6,55.5)	29.1 (27.8,30.4)	16.7 (15.7,17.8)	6.4 (5.8,7.2)
Malta	81.8 (80.3,83.3)	62.6 (60.7,64.4)	74.5 (72.8,76.2)	24.3 (22.7,26.0)	21.6 (20.0,23.2)	4.0 (3.3,4.9)
Netherlands	82.1 (81.0,83.2)	66.0 (64.6,67.3)	55.0 (53.5,56.4)	17.7 (16.6,18.8)	16.6 (15.5,17.7)	5.2 (4.6,5.9)
Norway	83.4 (82.0,84.6)	64.9 (63.2,66.6)	62.3 (60.6,64.1)	5.2 (4.5,6.1)	13.3 (11.6,15.2)	5.6 (4.5,6.9)
Poland	82.8 (81.8,83.8)	61.7 (60.4,63.1)	65.7 (64.4,67.0)	16.3 (15.3,17.3)	15.9 (14.9,16.9)	9.0 (8.2,9.8)
Portugal	89.7 (88.9,90.5)	54.1 (52.8,55.3)	66.7 (65.5,67.9)	15.2 (14.3,16.1)	18.0 (17.1,19.0)	5.1 (4.5,5.7)
Republic of Moldova	85.2 (84.1,86.2)	52.1 (50.7,53.5)	57.2 (55.8,58.6)	10.2 (9.3,11.1)	17.0 (15.9,18.1)	4.5 (3.9,5.1)
Romania	86.0 (84.9,87.0)	60.4 (58.9,61.8)	68.9 (67.5,70.2)	21.5 (20.3,22.7)	25.1 (23.9,26.4)	11.0 (10.1,12.0)
Russia	84.9 (83.7,85.9)	64.6 (63.2,66.0)	66.3 (64.9,67.7)	9.7 (8.8,10.6)	8.2 (7.4,9.1)	6.2 (5.5,7.0)
Scotland	82.5 (81.3,83.7)	64.3 (62.8,65.8)	63.6 (62.1,65.1)	17.2 (16.0,18.4)	18.4 (17.1,19.7)	4.5 (3.9,5.3)
Serbia	66.4 (64.9,67.9)	57.8 (56.2,59.3)	58.0 (56.4,59.5)	22.4 (21.1,23.8)	31.3 (29.9,32.8)	9.5 (8.6,10.5)
Slovakia	77.0 (75.7,78.1)	58.7 (57.3,60.1)	64.0 (62.6,65.4)	21.2 (20.1,22.5)	16.8 (15.7,17.9)	9.9 (9.1,10.9)
Slovenia	77.4 (76.3,78.5)	57.5 (56.2,58.8)	63.3 (62.0,64.5)	6.2 (5.6,6.9)	22.1 (21.1,23.2)	6.4 (5.8,7.1)
Spain	78.6 (77.4,79.8)	62.8 (61.3,64.2)	72.4 (71.1,73.7)	13.4 (12.4,14.4)	18.3 (17.1,19.5)	7.2 (6.4,8.0)
Sweden	86.0 (84.9,87.0)	73.4 (72.0,74.7)	56.7 (55.2,58.2)	5.0 (4.3,5.7)	11.1 (10.1,12.1)	6.1 (5.4,6.9)
Switzerland	85.6 (84.8,86.4)	54.3 (53.2,55.4)	54.4 (53.3,55.6)	20.2 (19.3,21.1)	17.9 (17.0,18.8)	6.3 (5.8,6.9)
Turkey	85.7 (84.8,86.6)	68.6 (67.4,69.8)	76.8 (75.7,77.8)	14.5 (13.6,15.4)	N/A	N/A
Ukraine	74.0 (72.9,75.1)	56.7 (55.5,57.9)	48.0 (46.8,49.2)	15.3 (14.4,16.2)	17.2 (16.3,18.2)	6.9 (6.3,7.6)
Wales	81.4 (80.7,82.0)	66.6 (65.8,67.3)	66.1 (65.3,66.8)	17.8 (17.2,18.4)	26.8 (26.1,27.6)	6.3 (5.9,6.7)
Total	81.1 (80.9,81.2)	59.9 (59.6,60.1)	61.2 (61.0,61.5)	15.7 (15.5,15.8)	19.2 (19.1,19.4)	6.9 (6.8,7.1)

Footnotes: Percentages are weighted for complex survey sample; N/A, data is unavailable.

Table S4: Prevalence of clusters of unhealthy behaviours by country, HBSC, 2018.

	**Prevalence of co-occurring unhealthy behaviours by countries (%)**						
**Countries**	**0**	**1**	**2**	**3**	**4**	**5**	**6**
**Non-European**							
Canada	12.0 (11.1,13.0)	28.3 (27.1,29.6)	23.1 (21.9,24.3)	28.1 (26.8,29.3)	6.8 (6.2,7.5)	1.6 (1.3,2.0)	0.1 (0.0,0.2)
Israel	3.4 (2.8,4.2)	23.8 (22.2,25.5)	24.9 (23.3,26.6)	31.7 (29.9,33.6)	12.6 (11.3,14.0)	3.2 (2.5,4.0)	0.3 (0.1,0.7)
Kazakhstan	9.2 (8.4,10.1)	21.6 (20.4,22.9)	29.5 (28.2,30.9)	34.1 (32.7,35.6)	4.7 (4.1,5.4)	0.8 (0.6,1.1)	0.0 (0.0,0.2)
**Eastern Europe**							
Armenia	5.8 (5.1,6.6)	23.2 (21.9,24.6)	32.6 (31.2,34.1)	27.9 (26.5,29.4)	8.0 (7.2,8.9)	2.1 (1.7,2.6)	0.3 (0.2,0.5)
Azerbaijan	4.3 (3.6,5.2)	16.5 (15.1,18.1)	21.2 (19.7,22.9)	50.7 (48.7,52.7)	6.5 (5.5,7.5)	0.7 (0.4,1.1)	0.0 (0.0,0.3)
Bulgaria	3.2 (2.7,3.8)	14.0 (13.0,15.0)	25.9 (24.6,27.2)	30.8 (29.4,32.1)	17.7 (16.6,18.9)	7.3 (6.6,8.1)	1.1 (0.9,1.5)
Czech Republic	5.7 (5.2,6.2)	20.2 (19.4,21.0)	22.0 (21.1,22.8)	33.1 (32.1,34.0)	14.0 (13.3,14.7)	4.2 (3.9,4.7)	0.9 (0.7,1.1)
Georgia	2.9 (2.4,3.5)	16.3 (15.2,17.5)	29.0 (27.6,30.5)	34.8 (33.4,36.4)	13.3 (12.3,14.4)	3.2 (2.7,3.8)	0.4 (0.3,0.7)
Hungary	3.9 (3.2,4.7)	12.8 (11.6,14.2)	20.5 (19.0,22.1)	38.6 (36.7,40.5)	16.5 (15.2,18.0)	6.2 (5.3,7.2)	1.4 (1.0,2.0)
Poland	4.4 (3.9,5.0)	17.4 (16.4,18.5)	22.7 (21.6,23.9)	38.7 (37.3,40.0)	12.2 (11.4,13.2)	3.9 (3.4,4.5)	0.7 (0.5,1.0)
Republic of Moldova	4.6 (4.1,5.3)	22.8 (21.5,24.0)	27.5 (26.2,28.9)	34.5 (33.1,35.9)	8.7 (7.9,9.5)	1.6 (1.3,2.1)	0.3 (0.2,0.5)
Romania	3.1 (2.6,3.7)	15.7 (14.6,16.9)	21.3 (20.0,22.6)	37.3 (35.8,38.8)	15.6 (14.5,16.8)	5.8 (5.1,6.6)	1.2 (0.9,1.6)
Russia	4.8 (4.2,5.6)	17.6 (16.4,18.8)	23.0 (21.7,24.4)	45.8 (44.2,47.4)	6.2 (5.4,7.0)	2.4 (2.0,3.0)	0.2 (0.1,0.4)
Slovakia	6.0 (5.3,6.7)	18.8 (17.7,20.0)	22.7 (21.5,24.0)	34.4 (33.0,35.8)	12.5 (11.5,13.5)	4.7 (4.1,5.4)	1.0 (0.7,1.3)
Ukraine	8.4 (7.7,9.1)	23.6 (22.6,24.7)	25.5 (24.4,26.6)	30.7 (29.6,31.9)	8.6 (7.9,9.3)	2.9 (2.5,3.4)	0.2 (0.2,0.4)
**Northern Europe**							
Denmark	3.8 (3.2,4.5)	20.8 (19.4,22.2)	26.0 (24.4,27.5)	36.0 (34.3,37.7)	9.8 (8.8,10.9)	3.3 (2.7,4.0)	0.4 (0.2,0.7)
England	5.8 (5.0,6.7)	21.6 (20.2,23.2)	23.0 (21.5,24.5)	35.9 (34.2,37.7)	10.7 (9.6,12.0)	2.3 (1.8,2.9)	0.7 (0.4,1.0)
Estonia	5.4 (4.8,6.1)	18.8 (17.7,19.9)	22.3 (21.1,23.5)	40.9 (39.5,42.4)	8.6 (7.8,9.5)	3.6 (3.1,4.2)	0.4 (0.2,0.6)
Finland	5.1 (4.1,6.2)	12.0 (10.5,13.6)	21.9 (20.0,23.9)	40.5 (38.2,42.8)	13.7 (12.1,15.3)	6.0 (5.0,7.2)	0.9 (0.5,1.4)
Iceland	8.7 (8.1,9.4)	22.2 (21.2,23.2)	19.1 (18.2,20.1)	44.1 (42.9,45.3)	4.3 (3.8,4.8)	1.4 (1.1,1.7)	0.2 (0.1,0.3)
Ireland	10.0 (9.0,11.0)	23.9 (22.6,25.4)	27.1 (25.6,28.5)	32.3 (30.8,33.8)	5.1 (4.4,5.8)	1.5 (1.1,1.9)	0.2 (0.1,0.4)
Latvia	3.3 (2.8,3.9)	12.4 (11.4,13.4)	24.9 (23.7,26.3)	41.8 (40.4,43.3)	12.3 (11.3,13.3)	4.7 (4.1,5.4)	0.4 (0.3,0.7)

Lithuania	5.0 (4.3,5.8)	16.8 (15.6,18.0)	21.1 (19.8,22.5)	39.3 (37.8,40.9)	11.5 (10.5,12.6)	5.3 (4.7,6.1)	0.9 (0.6,1.2)
Norway	4.1 (3.2,5.3)	18.0 (16.0,20.1)	22.3 (20.2,24.6)	44.3 (41.6,46.9)	8.4 (7.1,10.0)	2.3 (1.6,3.2)	0.7 (0.3,1.3)
Scotland	5.4 (4.7,6.2)	17.3 (16.1,18.6)	23.9 (22.5,25.3)	35.3 (33.7,36.8)	13.2 (12.1,14.3)	4.1 (3.5,4.8)	0.8 (0.6,1.2)
Sweden	3.8 (3.3,4.5)	17.6 (16.4,18.9)	27.9 (26.5,29.3)	40.7 (39.2,42.3)	6.9 (6.2,7.8)	2.7 (2.2,3.3)	0.3 (0.2,0.5)
Wales	4.7 (4.3,5.0)	15.3 (14.8,16.0)	21.1 (20.4,21.8)	36.4 (35.6,37.2)	16.5 (15.8,17.1)	5.2 (4.8,5.6)	0.9 (0.7,1.0)
**Southern Europe**							
Albania	6.7 (5.5,8.0)	25.3 (23.2,27.5)	30.6 (28.4,32.9)	27.1 (25.0,29.3)	7.8 (6.6,9.2)	2.1 (1.5,3.0)	0.4 (0.2,0.9)
Croatia	4.0 (3.4,4.5)	14.1 (13.2,15.1)	23.2 (22.0,24.4)	36.4 (35.0,37.8)	14.5 (13.5,15.5)	6.3 (5.7,7.1)	1.5 (1.2,1.9)
Greece	3.5 (2.9,4.1)	12.7 (11.7,13.8)	27.8 (26.4,29.3)	35.9 (34.3,37.4)	15.1 (13.9,16.3)	4.4 (3.8,5.1)	0.7 (0.5,1.0)
Italy	1.4 (1.1,1.8)	12.9 (11.9,14.0)	23.6 (22.3,24.9)	39.8 (38.3,41.4)	16.4 (15.3,17.6)	5.1 (4.4,5.8)	0.8 (0.6,1.1)
Macedonia	8.4 (7.6,9.3)	20.9 (19.8,22.2)	24.2 (22.9,25.5)	29.4 (28.1,30.8)	13.4 (12.4,14.5)	3.2 (2.8,3.8)	0.4 (0.3,0.7)
Malta	3.1 (2.5,3.8)	13.4 (12.1,14.9)	25.0 (23.3,26.7)	35.9 (34.0,37.8)	17.6 (16.1,19.2)	4.4 (3.6,5.3)	0.6 (0.4,1.0)
Portugal	2.9 (2.5,3.4)	18.1 (17.2,19.1)	26.2 (25.1,27.4)	37.1 (35.9,38.3)	11.7 (10.9,12.6)	3.3 (2.9,3.8)	0.6 (0.4,0.8)
Serbia	7.7 (6.9,8.6)	18.2 (17.0,19.5)	23.5 (22.1,24.9)	29.5 (28.1,31.0)	13.9 (12.8,15.0)	6.2 (5.5,7.1)	1.0 (0.7,1.3)
Slovenia	6.8 (6.1,7.4)	20.8 (19.7,21.9)	24.0 (22.9,25.2)	34.1 (32.8,35.3)	11.0 (10.2,11.8)	3.1 (2.7,3.6)	0.3 (0.2,0.5)
Spain	4.2 (3.7,4.9)	15.0 (13.9,16.1)	27.8 (26.5,29.2)	35.9 (34.5,37.4)	12.3 (11.3,13.3)	4.0 (3.4,4.6)	0.8 (0.5,1.1)
**Western Europe**							
Austria	5.4 (4.7,6.1)	18.2 (17.0,19.4)	24.8 (23.4,26.2)	34.0 (32.5,35.5)	12.6 (11.6,13.7)	4.5 (3.9,5.2)	0.6 (0.4,0.9)
Belgium (Flemish)	5.4 (4.7,6.1)	21.4 (20.2,22.7)	29.8 (28.4,31.2)	27.9 (26.6,29.3)	11.9 (10.9,12.9)	3.1 (2.6,3.7)	0.5 (0.3,0.8)
Belgium (French)	4.2 (3.7,4.8)	22.6 (21.5,23.8)	27.7 (26.5,29.0)	27.9 (26.6,29.1)	13.7 (12.8,14.7)	3.4 (2.9,3.9)	0.5 (0.3,0.7)
France	2.3 (2.0,2.7)	16.4 (15.5,17.3)	21.9 (20.8,22.9)	36.4 (35.2,37.6)	17.7 (16.8,18.6)	4.8 (4.3,5.3)	0.6 (0.5,0.9)
Germany	3.0 (2.6,3.6)	15.3 (14.3,16.5)	21.2 (20.0,22.5)	38.6 (37.1,40.1)	16.4 (15.3,17.6)	4.5 (3.9,5.2)	0.9 (0.6,1.2)
Luxembourg	3.6 (3.1,4.3)	18.1 (16.9,19.3)	22.4 (21.1,23.8)	35.9 (34.4,37.5)	14.7 (13.6,15.8)	4.1 (3.5,4.8)	1.2 (0.9,1.6)
Netherlands	4.1 (3.6,4.8)	17.9 (16.8,19.1)	29.1 (27.8,30.5)	33.3 (31.9,34.7)	11.6 (10.7,12.6)	3.4 (2.9,3.9)	0.5 (0.4,0.8)
Switzerland	4.7 (4.3,5.2)	22.2 (21.2,23.1)	24.8 (23.8,25.8)	31.7 (30.7,32.8)	12.4 (11.7,13.2)	3.4 (3.0,3.8)	0.8 (0.6,1.1)
Total	5.3 (5.2,5.4)	18.9 (18.7,19.1)	24.3 (24.1,24.5)	35.2 (35.0,35.4)	12.0 (11.8,12.1)	3.7 (3.6,3.8)	0.6 (0.6,0.6)

Footnotes: Prevalences are weighted for the survey sample.

Table S5: Prevalence of three or more unhealthy behaviours by sex using the 2018 HBSC survey.

**Countries**	**Overall**	**Male**	**Female**
Albania	37.4 (35.1,39.8)	45.9 (42.3,49.5)	30.6 (27.6,33.6)
Armenia	38.3 (36.8,39.8)	45.1 (42.8,47.3)	32.0 (30.0,34.1)
Austria	51.7 (50.1,53.3)	54.7 (52.4,56.9)	48.8 (46.6,51.0)
Azerbaijan	57.9 (55.9,59.8)	59.4 (56.5,62.3)	56.6 (53.9,59.3)
Belgium (Flemish)	43.4 (41.9,44.9)	47.5 (45.2,49.7)	39.6 (37.5,41.7)
Belgium (French)	45.4 (44.0,46.8)	47.5 (45.5,49.4)	43.4 (41.5,45.3)
Bulgaria	56.9 (55.5,58.4)	59.9 (57.8,61.9)	54.2 (52.2,56.2)
Canada	36.6 (35.2,37.9)	37.3 (35.4,39.3)	35.9 (34.1,37.8)
Croatia	58.7 (57.3,60.1)	61.2 (59.3,63.2)	56.2 (54.2,58.2)
Czech Republic	52.2 (51.2,53.2)	56.2 (54.8,57.6)	48.2 (46.8,49.6)
Denmark	49.5 (47.7,51.3)	51.7 (49.2,54.3)	47.4 (45.0,49.9)
England	49.6 (47.8,51.4)	52.2 (49.7,54.7)	46.9 (44.2,49.6)
Estonia	53.5 (52.1,55.0)	58.0 (55.9,60.0)	49.1 (47.1,51.2)
Finland	61.1 (58.8,63.3)	66.0 (62.7,69.1)	56.5 (53.2,59.6)
France	59.5 (58.3,60.7)	62.2 (60.4,63.9)	56.9 (55.2,58.6)
Georgia	51.8 (50.2,53.4)	55.2 (52.9,57.4)	48.6 (46.4,50.8)
Germany	60.4 (58.9,61.9)	64.1 (61.9,66.2)	57.2 (55.2,59.3)
Greece	56.0 (54.4,57.6)	57.5 (55.2,59.7)	54.6 (52.3,56.8)
Hungary	62.8 (60.9,64.6)	64.8 (61.9,67.5)	61.2 (58.7,63.7)
Iceland	50.0 (48.7,51.2)	52.6 (50.9,54.3)	47.4 (45.7,49.1)
Ireland	39.0 (37.4,40.6)	39.9 (37.7,42.2)	38.1 (35.9,40.4)
Israel	47.8 (45.9,49.8)	53.6 (50.7,56.5)	42.1 (39.6,44.7)
Italy	62.1 (60.6,63.6)	65.6 (63.5,67.7)	58.8 (56.7,60.9)
Kazakhstan	39.7 (38.2,41.2)	41.6 (39.5,43.7)	37.8 (35.8,39.9)
Latvia	59.3 (57.8,60.8)	60.8 (58.7,62.9)	57.9 (55.8,59.9)
Lithuania	57.1 (55.5,58.7)	60.5 (58.2,62.8)	53.7 (51.4,56.0)

Luxembourg	55.9 (54.3,57.5)	56.6 (54.4,58.9)	55.1 (52.9,57.4)
Macedonia	46.5 (45.0,48.0)	50.5 (48.4,52.7)	42.8 (40.7,44.8)
Malta	58.5 (56.5,60.5)	58.5 (55.6,61.3)	58.5 (55.8,61.2)
Netherlands	48.8 (47.3,50.3)	53.0 (50.9,55.1)	44.4 (42.3,46.4)
Norway	55.6 (52.9,58.2)	60.0 (56.3,63.7)	51.4 (47.7,55.1)
Poland	55.5 (54.1,56.8)	58.4 (56.5,60.4)	52.7 (50.7,54.6)
Portugal	52.7 (51.4,54.0)	54.5 (52.7,56.4)	51.0 (49.3,52.8)
Republic of Moldova	45.1 (43.6,46.5)	48.7 (46.6,50.8)	41.5 (39.5,43.6)
Romania	59.9 (58.3,61.4)	62.6 (60.4,64.8)	57.2 (55.0,59.4)
Russia	54.6 (53.0,56.2)	54.7 (52.3,57.0)	54.6 (52.3,56.8)
Scotland	53.4 (51.8,55.0)	58.1 (55.8,60.4)	49.1 (46.9,51.4)
Serbia	50.6 (49.0,52.2)	52.6 (50.3,55.0)	48.6 (46.4,50.9)
Slovakia	52.5 (51.0,54.0)	55.6 (53.6,57.7)	49.3 (47.2,51.4)
Slovenia	48.4 (47.1,49.7)	51.3 (49.5,53.2)	45.4 (43.5,47.3)
Spain	52.9 (51.4,54.5)	52.7 (50.5,54.9)	53.2 (51.0,55.3)
Sweden	50.7 (49.1,52.3)	52.4 (50.1,54.6)	49.1 (46.8,51.3)
Switzerland	48.4 (47.2,49.5)	52.8 (51.2,54.4)	43.8 (42.2,45.4)
Ukraine	42.5 (41.2,43.7)	44.6 (42.8,46.4)	40.3 (38.6,42.0)
Wales	58.9 (58.1,59.7)	60.3 (59.2,61.5)	57.5 (56.3,58.6)
Total	51.5 (51.3, 51.7)	54.5 (53.9, 54.6)	48.9 (48.6, 49.2)

Footnotes: Prevalences are weighted for the survey sample.

Table S6: Model comparison statistics and random effect parameters for mixed-effects logistic regression analysis of factors associated with three or more unhealthy behaviours among adolescents using the 2018 HBSC data.

Model group	Model type	LL	AIC	ICC-country level (%)	ICC-school level (%)	Variance (SE) at country level	Variance (SE) at school level	PCV - country level (%)	PCV - school level (%)
**Overall**	Null	-141618	283242	2.5	8.9	0.09 (0.01)	0.23 (0.01)	ref	ref
	Final model	-118383	236814	2	5.7	0.07 (0.01)	0.13 (0.01)	21.4	43
**Male**	Null	-69371	138749	2.4	8.2	0.08 (0.01)	0.20 (0.01)	ref	ref
	Final model	-57013	114073	2	5.9	0.07 (0.01)	0.13 (0.01)	24.4	16.1
**Female**	Null	-72414	144834	2.8	9.5	0.09 (0.01)	0.24 (0.01)	ref	ref
	Final model	-61504	123054	2.1	6.0	0.07 (0.01)	0.13 (0.01)	42.5	31.6

Footnotes: LL, Loglikelihood; AIC, Akaike Information criteria; ICC, Intra Class Correlation; PCV, Percentage Change in Variance; SE, Standard error.

Table S7: Mixed-effects logistic regression analysis of factors associated with ***two or more*** unhealthy behaviours among adolescents using HBSC 2018.

Fixed Effects	Overall<br>AOR (95% CI)	Male<br>AOR (95% CI)	Female<br>AOR (95% CI)
**Age**			
<15	1.00	1.00	1.00
≥15	1.79 (1.59,2.02) ***	1.87 (1.65,2.12) ***	1.76 (1.56,1.99) ***
**Gender**			
Male	1.28 (1.24,1.32) ***	-	-
Female		-	-
**Family affluence**			
Low	1.00	1.00	1.00
Medium	0.81 (0.78,0.84) ***	0.83 (0.78,0.89) ***	0.79 (0.75,0.82) ***
High	0.57 (0.54,0.60) ***	0.59 (0.56,0.64) ***	0.54 (0.51,0.57) ***
**Living with both parents**			
Yes	0.82 (0.78, 0.86) ***	0.83 (0.78,0.88) ***	0.80 (0.76, 0.85) ***
No	1.00	1.00	1.00
**Perceived family support**			
**Perceived family support**			
Low	1.00	1.00	1.00
Medium	0.89 (0.82,0.96) **	0.95 (0.88, 1.03)	0.83 (0.77,0.91) ***
High	0.60 (0.55,0.66) ***	0.63 (0.57,0.70) ***	0.58 (0.53, 0.63) ***
**Perceived peer support**			
**Perceived peer support**			
Low	1.00	1.00	1.00
Medium	0.97 (0.93, 1.01)	0.94 (0.89, 0.99) *	1.00 (0.94, 1.06)
High	0.81 (0.78,0.84) ***	0.73 (0.68, 0.77) ***	0.88 (0.84, 0.94) **
**School satisfaction**			
Not at all	1.00	1.00	1.00

Not very much	0.97 (0.93,1.01)	1.00 (0.94,1.06)	0.92 (0.85,0.99) *
Like a bit	0.72 (0.68,0.76) ***	0.74 (0.70,0.78) ***	0.68 (0.62,0.75) ***
Like a lot	0.46 (0.43,0.50) ***	0.48 (0.45,0.52) ***	0.44 (0.40,0.48) ***
**School pressure**			
Not at all	1.00	1.00	1.00
A little	1.29 (1.24,1.34) ***	1.29 (1.23,1.36) ***	1.29 (1.21,1.37) ***
Some	1.35 (1.27,1.43) ***	1.32 (1.25,1.40) ***	1.37 (1.25,1.50) ***
A lot	1.21 (1.11,1.32) ***	1.16 (1.08,1.26) ***	1.24 (1.10,1.39) ***
**School-level mean family affluence**	0.98 (0.94,1.03)	0.98 (0.93, 1.02)	0.97 (0.93,1.02)
**Subregion**			
Western Europe	1.00	1.00	1.00
Eastern Europe	0.99 (0.68,1.42)	0.98 (0.69,1.40)	0.97 (0.66,1.42)
Northern Europe	0.89 (0.70,1.13)	0.92 (0.72,1.18)	0.87 (0.68,1.10)
Southern Europe	1.06 (0.76,1.47)	1.03 (0.74,1.42)	1.05 (0.74,1.50)

Footnotes: Model adjusted for individual, school, and country-level variables included in the model plus Human Development Index (HDI); AOR, adjusted odds ratio; *** p<0.0001; **p<0.01; *p<0.05.

Table S8: Mixed-effects logistic regression analysis of factors associated with ***four or more*** unhealthy behaviours among adolescents using HBSC 2018.

Fixed Effects	Overall	Male	Female
	**AOR (95% CI)**	**AOR (95% CI)**	**AOR (95% CI)**
**Age**			
**<15**			
≥15	3.22 (2.93,3.53) ***	3.22 (2.94,3.53) ***	3.22 (2.94,3.53) ***
**Gender**			
Male	1.27 (1.19,1.35) ***	-	-
Female		-	-
**Family affluence**			
Low	1.00	1.00	1.00
Medium	0.94 (0.90,0.99) *	0.94 (0.89,1.00) *	0.94 (0.87,1.00)
High	0.88 (0.83,0.93) ***	0.89 (0.82,0.97) **	0.83 (0.78,0.89) ***
**Living with both parents**			
Yes	0.75 (0.72, 0.79) ***	0.77 (0.74,0.81) ***	0.75 (0.70,0.80) ***
No			
**Perceived family support**			
Low	1.00	1.00	1.00
Medium	0.73 (0.70, 0.76) ***	0.74 (0.70, 0.79) ***	0.72 (0.68, 0.76) ***
High	0.51 (0.48, 0.55) ***	0.56 (0.52, 0.60) ***	0.47 (0.43, 0.50) ***
**Perceived peer support**	1.05 (1.02,1.07) ***	1.02 (1.00,1.05)	1.07 (1.03,1.11) ***
**Perceived peer support**			
Low	1.00	1.00	1.00
Medium	1.05 (1.01,1.10) *	1.03 (0.98, 1.08)	1.06 (0.99, 1.14)
High	1.13 (1.07, 1.19) ***	1.06 (1.00, 1.13) *	1.18 (1.10,1.27) ***
**School satisfaction**			
Not at all			

Not very much	0.73 (0.69,0.77) ***	0.75 (0.70,0.80) ***	0.70 (0.64,0.75) ***
Like a bit	0.48 (0.44,0.52) ***	0.51 (0.46,0.56) ***	0.44 (0.40,0.49) ***
Like a lot	0.31 (0.28,0.36) ***	0.35 (0.31,0.40) ***	0.27 (0.23,0.32) ***
**School pressure**			
Not at all			
A little	1.09 (1.02,1.16) **	1.07 (1.00,1.14)	1.12 (1.03,1.23) **
Some	1.15 (1.07,1.23) ***	1.14 (1.05,1.24) **	1.17 (1.06,1.28) ***
A lot	1.20 (1.10,1.32) ***	1.18 (1.07,1.30) ***	1.20 (1.07,1.35) **
**School-level mean family affluence**	1.03 (0.96,1.11)	1.02 (0.95,1.09)	1.04 (0.96,1.12)
**Subregion**			
Western Europe	1.00	1.00	1.00
Eastern Europe	0.91 (0.56,1.48)	0.84 (0.52,1.35)	0.98 (0.59,1.65)
Northern Europe	0.61 (0.42,0.87) **	0.57 (0.40,0.82) **	0.63 (0.43,0.93) *
Southern Europe	1.02 (0.71,1.48)	0.93 (0.65,1.33)	1.11 (0.74,1.68)

Footnotes: Model adjusted for individual, school, and country-level variables included in the model plus Human Development Index (HDI); AOR, adjusted odds ratio; *** p<0.0001; **p<0.01; *p<0.05.

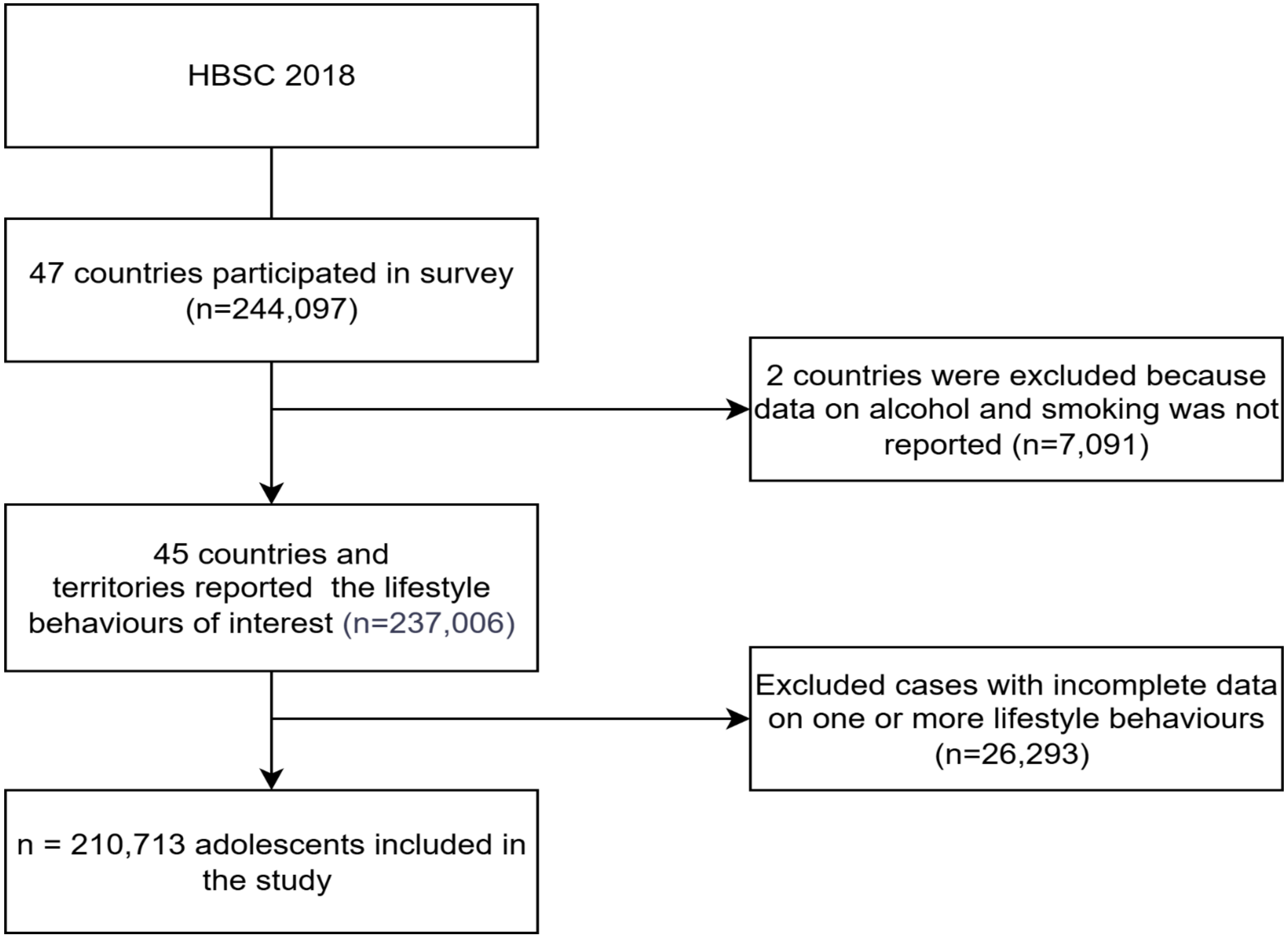

Figure S1: Flow chart illustrating the sample selection process using the 2018 HBSC.

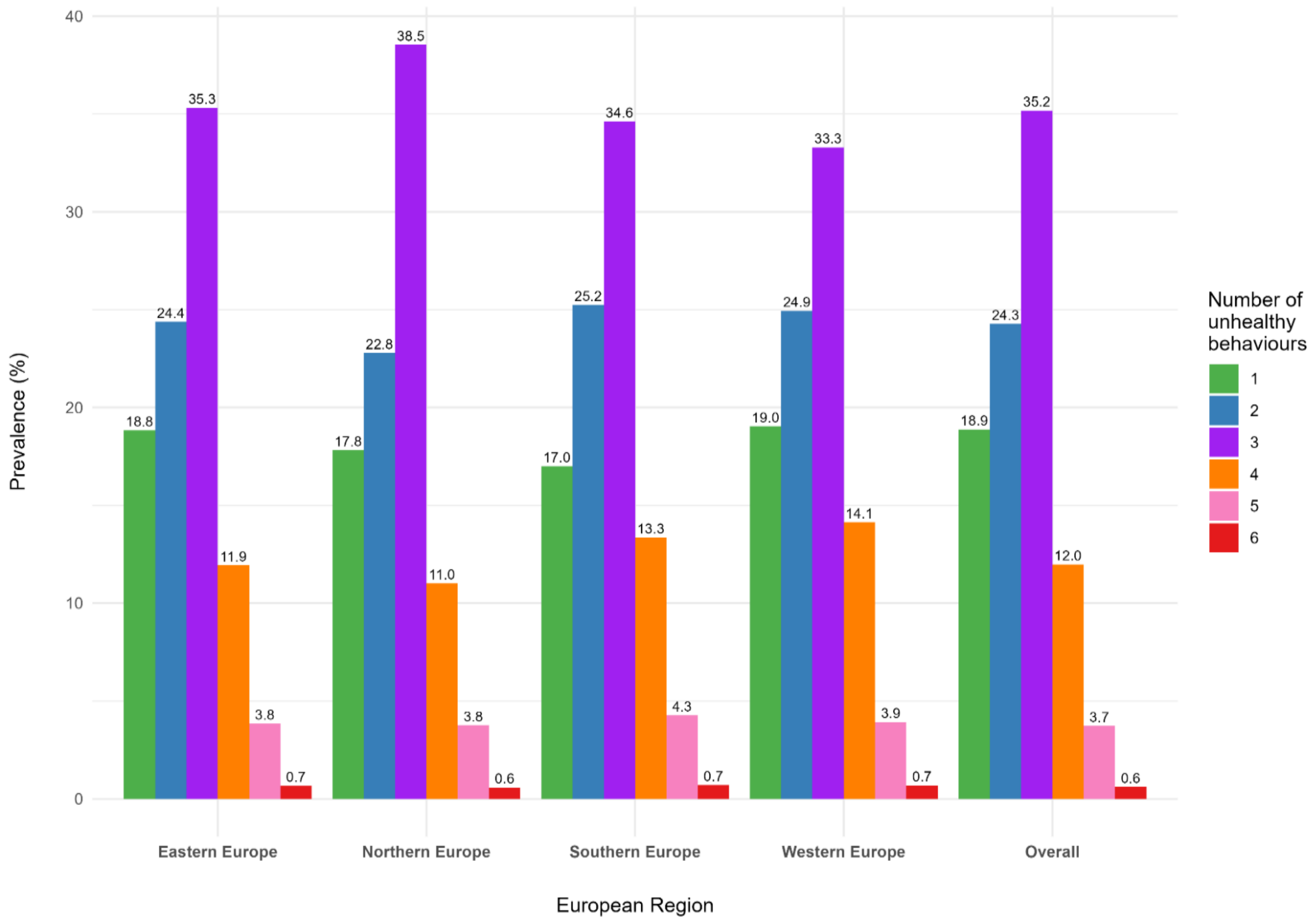

Figure S2: Prevalence of one or more unhealthy behaviours among adolescents by European regions, HBSC, 2018.

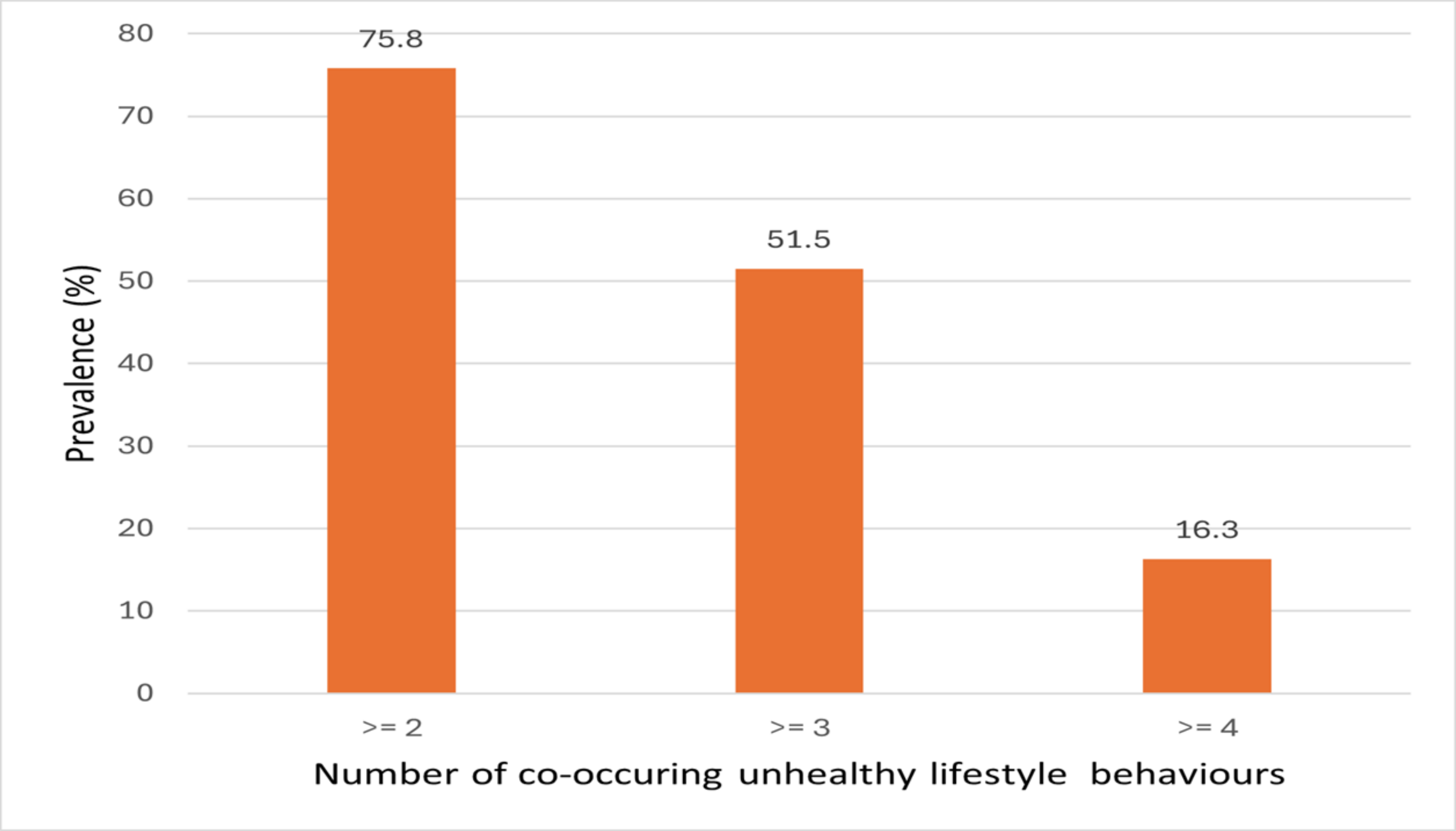

Figure S3: Prevalence of co-occurring unhealthy lifestyle behaviours among adolescents, HBSC, 2018.

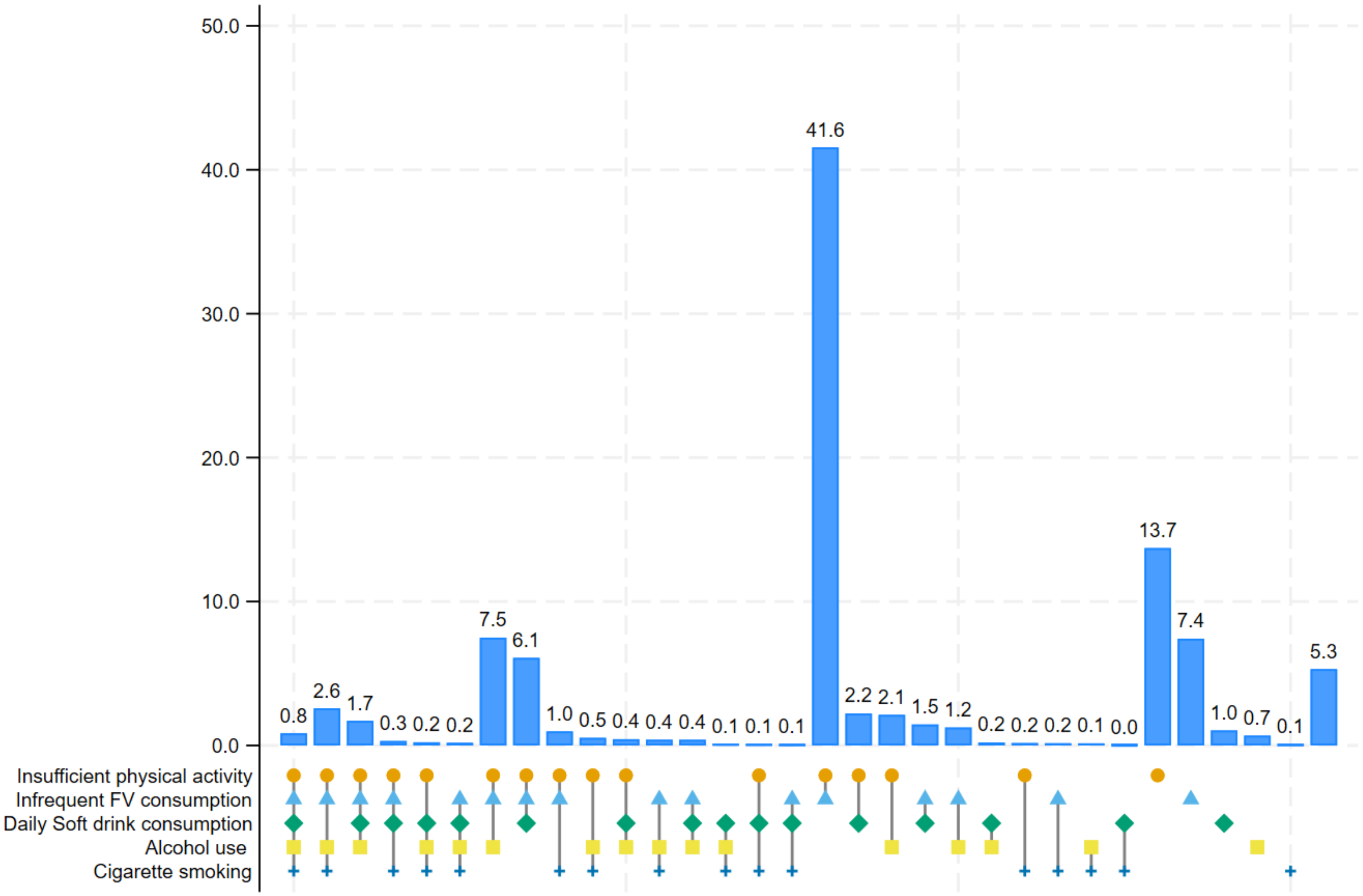

Figure S4: Clustering of Unhealthy Lifestyle behaviours among adolescents, HBSC, 2018.

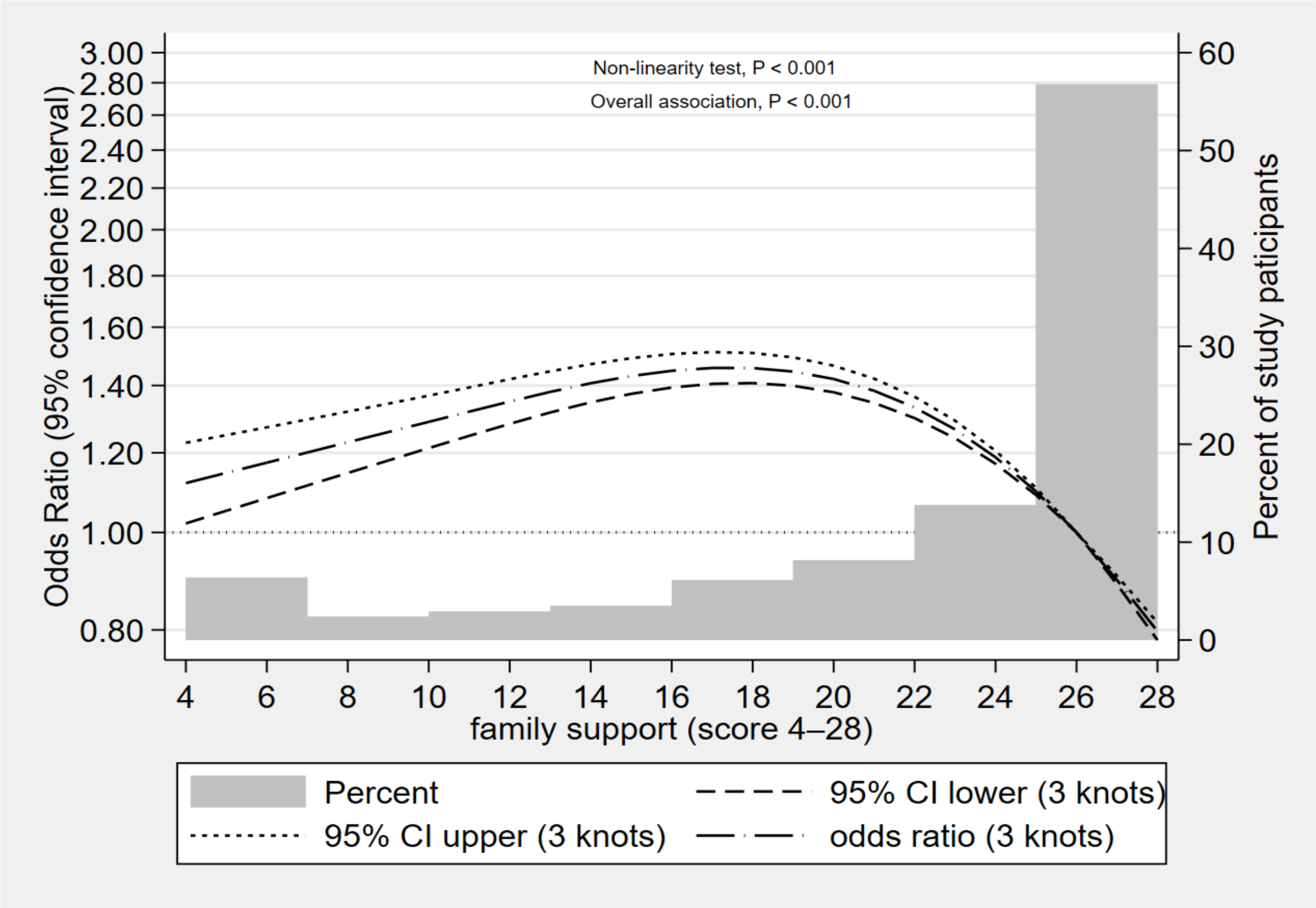

Figure S5: Non-linear association between family support and the odds of three or more unhealthy behaviours among adolescents. Odds ratios (dashed line) and 95% confidence intervals (upper and lower dashed lines) were estimated using restricted cubic splines with 3 knots from the fully adjusted model*. The reference value (OR = 1) corresponds to the median peer support score of 26. Grey bars represent the distribution of participants (right axis).

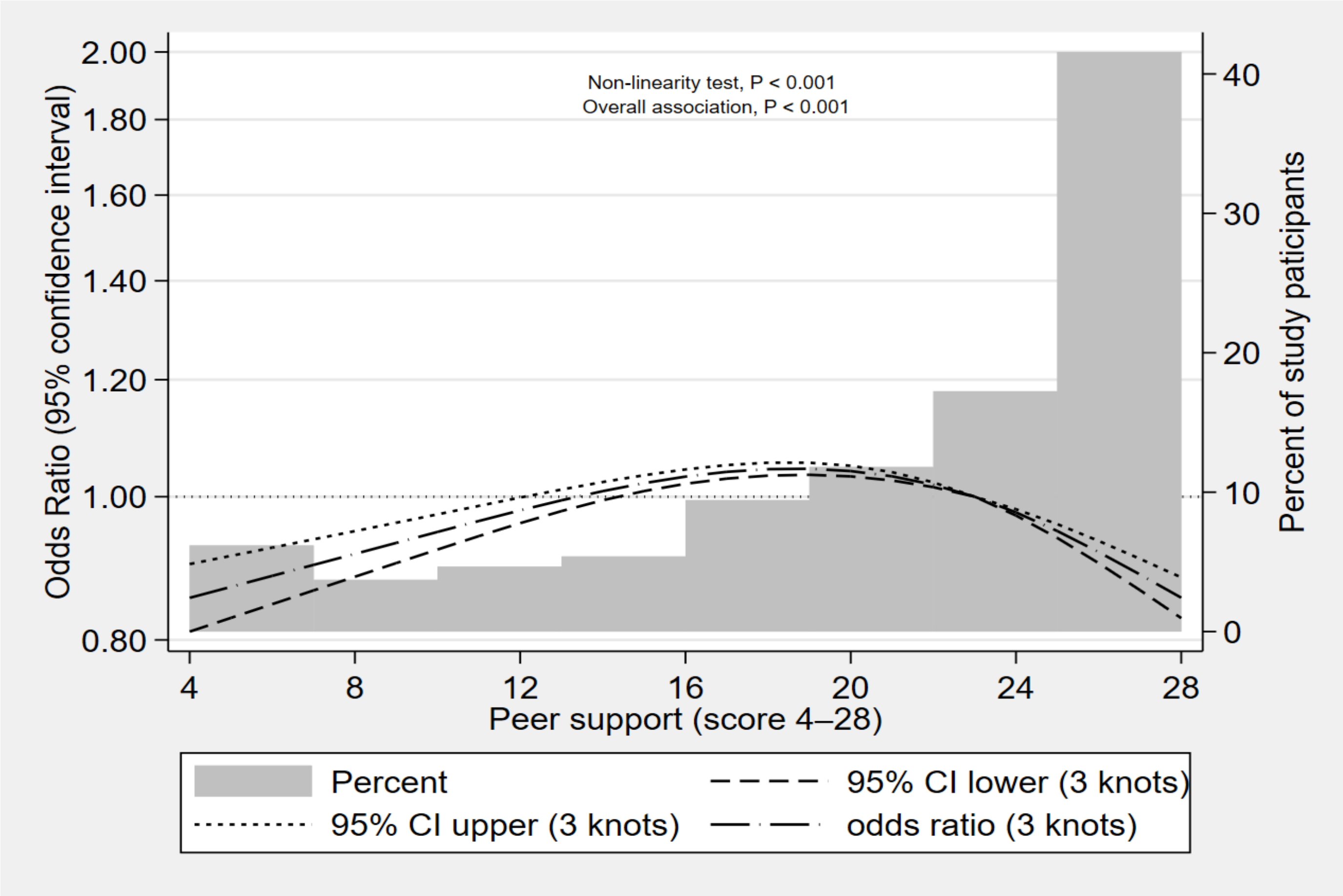

Figure S6: Non-linear association between peer support and the odds of three or more unhealthy behaviours among adolescents. Odds ratios (dashed line) and 95% confidence intervals (upper and lower dashed lines) were estimated using restricted cubic splines with 3 knots from the fully adjusted model*. The reference value (OR = 1) corresponds to the median peer support score of 23. Grey bars represent the distribution of participants (right axis).
